# Comparative proteomics of related symbiotic mussel species reveals high variability of host–symbiont interactions

**DOI:** 10.1038/s41396-019-0517-6

**Published:** 2019-11-04

**Authors:** Ruby Ponnudurai, Stefan E. Heiden, Lizbeth Sayavedra, Tjorven Hinzke, Manuel Kleiner, Christian Hentschker, Horst Felbeck, Stefan M. Sievert, Rabea Schlüter, Dörte Becher, Thomas Schweder, Stephanie Markert

**Affiliations:** 1 Institute of Pharmacy, University of Greifswald, Greifswald, Germany; 2 European Molecular Biology Laboratory, Meyerhofstrasse 1, 69117 Heidelberg, Germany; 3 Max Planck Institute for Marine Microbiology, Bremen, Germany; 4 Quadram Institute of Bioscience, Norwich, UK; 5 Institute of Marine Biotechnology, Greifswald, Germany; 6 Department of Plant and Microbial Biology, North Carolina State University, Raleigh, NC USA; 7 Institute of Microbiology, University of Greifswald, Greifswald, Germany; 8 Scripps Institution of Oceanography, La Jolla, CA USA; 9 Woods Hole Oceanographic Institution, Woods Hole, MA USA; 10 Imaging Center of the Department of Biology, University of Greifswald, Greifswald, Germany

**Keywords:** Microbial ecology, Water microbiology, Symbiosis, Bacterial physiology, Proteomics

## Abstract

Deep-sea *Bathymodiolus* mussels and their chemoautotrophic symbionts are well-studied representatives of mutualistic host–microbe associations. However, how host–symbiont interactions vary on the molecular level between related host and symbiont species remains unclear. Therefore, we compared the host and symbiont metaproteomes of Pacific *B. thermophilus*, hosting a thiotrophic symbiont, and Atlantic *B. azoricus*, containing two symbionts, a thiotroph and a methanotroph. We identified common strategies of metabolic support between hosts and symbionts, such as the oxidation of sulfide by the host, which provides a thiosulfate reservoir for the thiotrophic symbionts, and a cycling mechanism that could supply the host with symbiont-derived amino acids. However, expression levels of these processes differed substantially between both symbioses. Backed up by genomic comparisons, our results furthermore revealed an exceptionally large repertoire of attachment-related proteins in the *B. thermophilus* symbiont. These findings imply that host–microbe interactions can be quite variable, even between closely related systems.

## Introduction

*Bathymodiolus* mussels harbor chemosynthetic bacterial symbionts in their gills and thrive in diverse marine habitats worldwide [1–3]. The intracellular symbionts fix dissolved inorganic carbon into organic compounds using the oxidation of reduced chemicals, such as methane, H_2_S, short-chain alkanes, or hydrogen, as energy source [4–7]. *Bathymodiolus* symbioses show a high degree of host–symbiont specificity, i.e., each host species harbors one (or several) distinct symbiont phylotype(s) [8]. *B. thermophilus*, for example, which colonizes hydrothermal vent fields on the East Pacific Rise (EPR), hosts a thiotrophic (sulfur-oxidizing, SOX) symbiont [9, 10]. In contrast, *B. azoricus* from the Mid-Atlantic Ridge (MAR) contains two symbiont phylotypes, a SOX symbiont (thiotroph) and a methane-oxidizing (MOX) symbiont (methanotroph) [5]. Despite these differences, *B. thermophilus* and *B. azoricus* are phylogenetically closely related [1, 2], and their thiotrophic symbionts, too, show close phylogenetic proximity [11, 12].

Recently, we reported a number of physiological interactions between host and symbionts in *B. azoricus* that provide metabolic integrity to the symbiosis as a whole [13]. However, little is known about these interactions in other *Bathymodiolus* host–symbiont combinations. Our current study therefore aims to identify similarities and specific differences in metabolic and physical interactions in the two geographically distant *Bathymodiolus* species *B. thermophilus* and *B. azoricus*.

## Methods

All methods are described in detail in the Supplementary Material. Briefly, for proteomic analyses, three *B. thermophilus* individuals were collected from the Tica vent field on the EPR at 9°50.39′N, 104°17.49′W in 2511 m water depth, and three *B. azoricus* specimens were collected from the Menez Gwen vent field on the MAR at 37°50′41′′N, 31°31′10′′W in 860 m water depth. The bivalves were dissected on board, and gills and foot tissue samples were separately frozen immediately. In addition, symbiont and host fractions were enriched from gill homogenate by differential centrifugation and/or gradient centrifugation [14] and enrichment was confirmed by CARD-FISH analyses. The soluble proteome was extracted from all sample types. To enhance identification of symbiont membrane proteins, which could be involved in host interactions, we additionally extracted the membrane proteome of gill samples (both hosts) and enriched symbiont samples (*B. azoricus*). Supplementary Table S1a shows an overview of all sample types and replicate numbers analyzed in this study. Mass spectrometric analyses were performed using an LTQ-Orbitrap Velos mass spectrometer and/or an LTQ-Orbitrap Classic mass spectrometer (both Thermo Fisher, Bremen, Germany). MS/MS spectra were searched against an in-house compiled comprehensive target-decoy database containing protein sequences of *Bathymodiolus* symbionts and host. Normalized spectral abundance factors were calculated as a measure of relative protein abundance in each sample (%NSAF) and for each organism (%OrgNSAF). Significant abundance differences between (a) thiotrophic symbiont protein orthologs in *B. thermophilus* and *B. azoricus*, and (b) different *B. thermophilus* sample types were determined using a Welch’s *t*-test with permutation-based false discovery rate of 5%. To support our proteomic observations, we conducted comparative genome analyses, which included four thiotrophic *Bathymodiolus* symbionts (of *B. thermophilus*, *B. azoricus*, *B. septemdierum*, and *Bathymodiolus*. sp.), two thiotrophic clam symbionts (“*Candidatus* Ruthia magnifica” and “*Candidatus* Vesicomyosocius okutanii”) and two free-living SOX bacteria (“*Candidatus* Thioglobus autotrophicus” and “*Candidatus* Thioglobus singularis”), whose genomes were obtained from GenBank and IMG (Supplementary Table S1b). The protein sequence database and all proteome raw data are available via PRIDE [15] with the dataset identifier PXD011639.

## Results and discussion

**Fig. 1:**
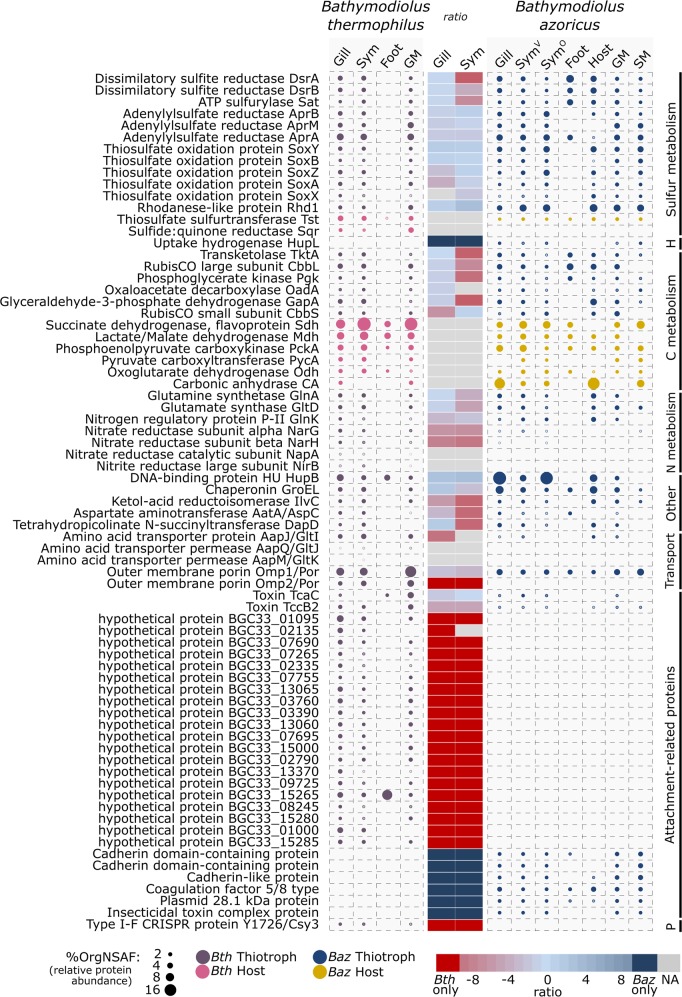
Relative abundance of proteins in major metabolic categories in *B. thermophilus* (*Bth*) and *B. azoricus* (*Baz*). Bubble size corresponds to protein abundance in %OrgNSAF (average values, for replicate numbers see Supplementary Table S1a; see Supplementary Tables S2 and S3 for a complete list of all identified proteins). Sample types: we analyzed the soluble proteome of symbiont-containing whole gill tissue (Gill) and symbiont-free foot tissue (Foot). In addition, we selectively enriched symbiont fractions (symbiont cell pellet, Sym) and host proteins (host-enriched supernatant, Host, *Baz* only) from gill tissue using gradient centrifugation, and analyzed their soluble proteome. For enhanced identification of membrane-associated symbiont proteins, we additionally analyzed the membrane proteome of whole gill tissue samples (gill membrane fraction, GM) and enriched symbionts (symbiont membrane fraction, SM, *Baz* only). *Baz* Sym samples were analyzed in an LTQ-Orbitrap Velos (V) mass spectrometer and in an LTQ-Orbitrap Classic (O) mass spectrometer. The heat map in the center shows ratios of symbiont protein abundance in *B. thermophilus* and *B. azoricus* Gill and Sym samples (Velos measurements only). Ratios were calculated from CLR-transformed %OrgNSAF values (see Supplementary Methods). Negative ratios (red cells) indicate higher abundance in *B. thermophilus*, while positive ratios (blue cells) indicate higher abundance in *B. azoricus*. Gray cells (NA) indicate proteins that were either not compared, or that lacked the minimum number of valid values for reliable ratio calculations (see also Supplementary Table S4). Major metabolic categories are indicated on the right. H hydrogen oxidation, P phage defense

Our metaproteome analysis of two *Bathymodiolus* symbioses provided a detailed picture of individual metabolic processes and hitherto unknown interactions between all symbiotic partners (Fig. 1). The most prominent similarities and differences observed between *B. azoricus* and *B. thermophilus* are outlined below (for an overview of total protein identifications in all sample types see Supplementary Results I).

**Fig. 2:**
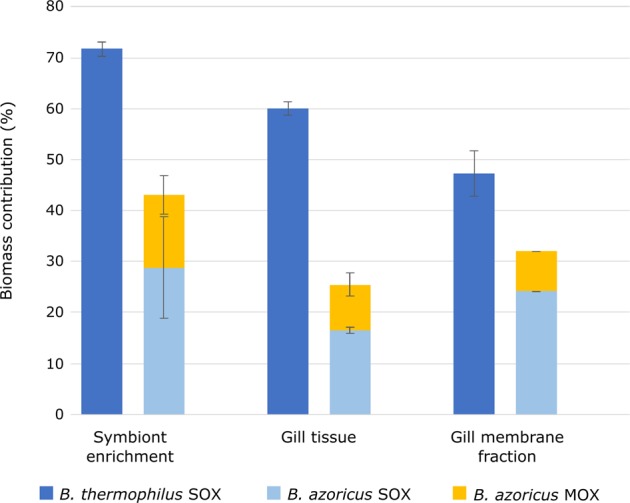
Biomass contributions of symbionts in *B. thermophilus* and *B. azoricus*. Total symbiont biomass was substantially higher in *B. thermophilus* than in *B. azoricus* in whole gill tissue as well as in enriched symbiont fractions and in gill membrane fractions. Biomass contributions were calculated from the total number of spectra recorded for each organism during MS/MS analyses [16]. Error bars indicate standard deviations (all *B. thermophilus* samples: *n* = 3; *B. azoricus* enriched symbiont fraction and whole gill tissue: *n* = 2; *B. azoricus* gill membrane fraction: two biological replicates were pooled for MS analysis). SOX sulfur-oxidizing symbiont, MOX methane-oxidizing symbiont

**Fig. 3:**
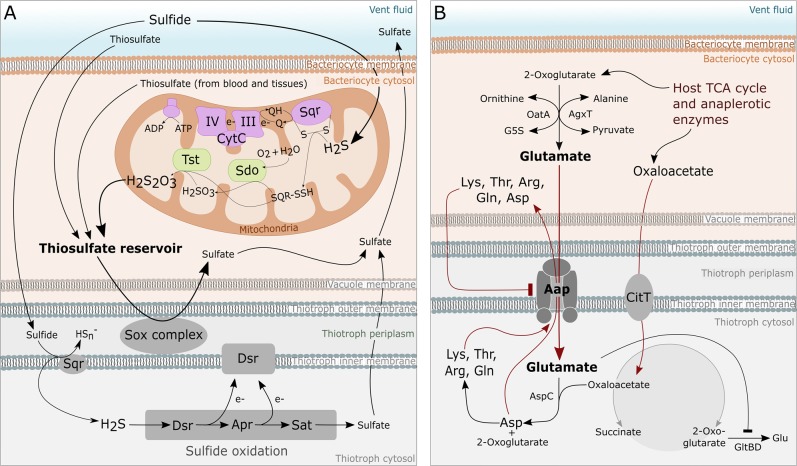
Metabolic interactions in *Bathymodiolus* mussels. **a** Thiosulfate generated by mitochondrial sulfide oxidation may accumulate in host tissues and could be used as an energy source by the thiotrophic symbiont. Purple: host mitochondrial membrane-associated enzymes. Green: host mitochondrial matrix enzymes. Gray: thiotrophic symbiont enzymes. Tst thiosulfate sulfurtransferase, Sdo sulfur dioxygenase, Sqr sulfide:quinone reductase, III coenzyme Q complex of respiratory chain, IV cytochrome c oxidase complex, Dsr dissimilatory sulfite reductase complex, Apr adenylylsulfate reductase complex, Sat ATP sulfurylase. Please note that sulfate and thiosulfate transport across host and symbiont membranes involves transporter proteins, which are not shown in this figure, because their identities and exact functions are yet unclear. **b** Proposed model of amino acid cycling between host and thiotrophic symbionts in *Bathymodiolus*. The symbiont’s general l-amino acid ABC transporter Aap imports host glutamate and exports aspartate (and presumably other amino acids) synthesized by the symbiont. Red arrows indicate amino acid biosynthetic routes that are shared between host and symbiont, whereas black indicates routes that are exclusive to the host or the symbiont. Arrows with flat ends suggest an inhibitory action. OatA: host ornithine aminotransferase, AgxT: host alanine aminotransferase, AspC: symbiont aspartate transaminase, GltBD: symbiont glutamate synthase, CitT: symbiont citrate transporter, Dct: symbiont tripartite ATP-independent periplasmic transporter. Lys, Thr, Arg, Gln, Asp: lysin, threonine, arginine, glutamine, aspartate; G5S: l-glutamate 5-semialdehyde

(1) Total symbiont biomass was substantially higher in *B. thermophilus* than in *B. azoricus* (Fig. 2). While the SOX symbiont population of *B. thermophilus* contributed 60% of total gill biomass, the total symbiont population of *B. azoricus* contributed only 25.3% (SOX: 16.4%, MOX: 8.9%, calculated based on protein abundance [16], Supplementary Table S8). This suggests that *B. thermophilus* may acquire a higher proportion of its nutrition through its symbionts than *B. azoricus*, in which filter-feeding might play a more prominent role. Previous findings based on the degree of convolution in the digestive tract in both mussels [17] and on the incorporation of dissolved and particulate organic matter in *B. azoricus* [18] support this idea. *B. thermophilus* specimens in our study were sampled in notably greater water depth (2511 m) and thus probably had access to less sinking biomass for filter-feeding than *B. azoricus* specimens (860 m depth). As thiotrophic and methanotrophic symbionts supposedly contribute equally to *B. azoricus*’ nutrition (as suggested for *Bathymodiolus* sp. [19]), the presence of the methanotroph likely does not counterbalance the lower total symbiont biomass, indicating that *B. azoricus* may indeed receive less nutrients from its symbiont population than *B. thermophilus*. The relative contributions of symbiont-derived nutrition and filter-feeding in *B. azoricus* appear to vary with season and physiological host factors such as mussel size [20–22]. We can therefore not rule out that dissimilar specimen sizes and sampling dates for *B. thermophilus* and *B. azoricus* (see Supplementary Methods) may have influenced our results, but we assume that this potential effect is negligible.

(2) Both *Bathymodiolus* hosts appear to oxidize sulfide and provide a thiosulfate reservoir for their symbionts. We identified a host sulfide:quinone reductase (Sqr) homolog (BAGiLS_015482, 61% sequence identity to mitochondrial sulfide:quinone oxidoreductase of the copepod *Eurytemora affinis*) in *B. thermophilus*, and a host sulfurtransferase (BAGiLS_000284, 53.8% identity to sulfurtransferase of the Pacific oyster *Crassostrea gigas*) in *B. thermophilus* and *B. azoricus* (Fig. 1, Supplementary Tables S2 and S3). Both are involved in the mitochondrial oxidation of sulfide to thiosulfate (Fig. 3a). They were enriched or exclusively detected in symbiont-containing samples compared with symbiont-free foot samples, indicating that mitochondrial sulfide oxidation is particularly relevant near the symbionts. As an inhibitor of aerobic respiration, hydrogen sulfide is toxic to aerobic organisms [23]. Invertebrate hosts of thiotrophic bacteria have therefore developed various strategies to shield their tissues from sulfide toxicity [24, 25], including the oxidation of sulfide into less harmful sulfur forms [26]. Our results strongly support the idea that *B. thermophilus* turns toxic sulfide into the less toxic thiosulfate by mitochondrial sufide oxidation, which may effectively function as a means of sulfide detoxification. This concept was first described for the thiotrophic symbiont-hosting clam *Solemya reidi* [27], but has since been reported for various other symbiotic and nonsymbiotic animals, including *Bathymodiolus* species [28–30].

The thiotrophic symbionts of *B. thermophilus and B. azoricus* use thiosulfate as an energy source [13, 31]. Proteins required for this thiosulfate oxidation process, i.e., the Sox multienzyme complex, showed quite similar total abundances in both thiotrophic symbionts in this study, with 2.03 %OrgNSAF in gill tissue in *B. azoricus* and 1.98% in *B. thermophilus* (Fig. 1, Supplementary Tables S2–S4). This suggests that both symbionts experience comparable thiosulfate levels in their microhabitat, the gill tissue, although their macro-environments differ with respect to host species and geographic location. As previously suggested [31, 32], mitochondrial sulfide oxidation in *Bathymodiolus* gills may thus create a pool of thiosulfate, which provides a stable energy source for the thiotrophic symbionts.

(3) We identified several copies of the host enzyme carbonic anhydrase (CA) with significantly higher abundances in symbiont-containing samples than in foot tissue samples in both *Bathymodiolus* hosts, indicating the involvement of these enzymes in symbiosis-related processes (Fig. 1, Supplementary Fig. S2). CAs interconvert HCO_3_^−^ and CO_2_, turning the diffusible gas CO_2_ into a nondiffusible form (and back). The two CA homologs BAGiLS_000922 and BAGiLS_000924 were the most abundant proteins in *B. azoricus* gill samples (5.2 %OrgNSAF) and host-enriched gill supernatant samples (6.9 %OrgNSAF; Supplementary Table S3, Fig. 1). In contrast, while three CAs were detected in *B. thermophilus* symbiont-containing samples (BAGiLS_000922, BAGiLS_000924. BAGiLS_003177), their total abundance was about 100-fold lower (0.052 %OrgNSAF in gills, 0.066 %OrgNSAF in enriched symbiont samples, Supplementary Table S2) than in *B. azoricus*. We hypothesize that the high expression of host CA in *B. azoricus* may be a response to CO_2_ released by the methanotrophic symbiont as end-product of methane oxidation. Possibly, CA in gill tissue may convert this methanotroph-derived CO_2_ to HCO_3_^−^, thus immobilizing and concentrating it for efficient fixation by the thiotroph. A function of abundant host CA in providing chemoautotrophic symbionts with inorganic carbon has been suggested for several marine invertebrates, including various *Bathymodiolus* species, *Calyptogena* species, and *Riftia pachyptila* [33–35]. In *B. thermophilus*, which lacks a methanotrophic symbiont, CO_2_ concentrations might be lower, which would require lower CA abundance, compared with *B. azoricus*. Both hosts thus appear to regulate their enzyme repertoire according to the specific requirements of their respective symbionts (Supplementary Discussion II, Supplementary Fig. S2).

(4) An amino acid cycling mechanism could provide *Bathymodiolus* hosts with symbiont-derived amino acids and appears to be particularly relevant in *B. thermophilus*. We detected a broad specificity l-amino acid ABC transporter (AapJQMP) in both *Bathymodiolus* SOX symbiont proteomes, which could be involved in selective “leakage” of symbiont amino acids to the host (Fig. 3b). Aap has a preference for polar amino acids and acts not only as an uptake transporter, but—in the presence of extracellular amino acids—also as an efflux transporter [36, 37]. In the well-studied *Rhizobium* symbiosis, Aap was shown to enable the cycling of amino acids between the plant host and root bacteroids [38, 39]. The glutamate-generating host enzymes ornithine aminotransferase (OatA: BAGiLS_006873, BAGiLS_004723) and alanine aminotransferase (AgxT: BAGiLS_022026) were notably more abundant or even exclusively detected in symbiont-containing samples compared with foot tissue in both *Bathymodiolus* hosts (Supplementary Tables S2 and S3). All identified peptides were unique to the host proteins and were not shared with any symbiont proteins. These proteins could produce glutamate in the direct vicinity of the symbionts for uptake by the bacterial Aap transporter. After import through Aap, glutamate could be transaminated in the bacterial cytoplasm by the symbiont's aspartate aminotransferase (AspC: OIR24744.1, SEH69114.1), which we identified in both thiotrophic symbionts, and the resulting aspartate could be recycled into the *Bathymodiolus* bacteriocyte. A similar amino acid cycling strategy was described in the *Buchnera*-aphid symbiosis [40]. Other amino acids besides aspartate and glutamate might also be cycled, as proposed for *Rhizobium* [38]. This mechanism would allow the *Bathymodiolus* host to compensate for its previously proposed inability to synthesize aspartate and many other amino acids autonomously ([13], Supplementary Table S5) by harnessing the symbiont’s biosynthetic machinery (see also Supplementary Discussion III). Simultaneously, both *B. azoricus* and *B. thermophilus* seem to supply their respective thiotrophic symbionts with oxaloacetate, an essential intermediate the bacteria cannot synthesize on their own ([13], this study; Fig. 3b). Close metabolic interdependency thus seems to be a typical feature of *Bathymodiolus* symbioses.

Interestingly, Aap was considerably more abundant in the *B. thermophilus* symbiont (the periplasmic solute-binding subunit AapJ, OIR25769.1, alone contributed ~1% of the entire symbiont proteome, Fig. 1), than in the *B. azoricus* thiotroph (SEH78249.1, <0.1 %OrgNSAF in the symbiont fraction). Possibly, this may be because *B. thermophilus* obtains a relatively larger part of its nutrition from its symbionts than *B. azoricus* (see above).

(5) Symbiont attachment-related proteins (ARPs) were highly abundant in *B. thermophilus* and may be involved in interactions with the host. We detected a large set of 129 *B. thermophilus* symbiont proteins involved in surface-binding and cell-cell adhesion, which together made up 23.9% of the symbiont’s proteome in gill tissue (Supplementary Table S6b). Most of these proteins (126) are predicted to be either attached to the symbiont cell surface or secreted into the surrounding host vacuole, and 127 were more abundant in gill samples (gill and/or gill membrane) than in symbiont-enriched fractions. The *B. azoricus* thiotroph, on the other hand, expressed only 16 ARPs, accounting for 3.5 %OrgNSAF in gill samples (Supplementary Table S6c). To judge whether the high number of ARPs observed in the *B. thermophilus* thiotroph poses an exception or rather a common feature of thiotrophic *Bathymodiolus* symbionts, we compared the *B. thermophilus* symbiont’s genome to the genomes of three other thiotrophic *Bathymodiolus* symbionts, two thiotrophic clam symbionts, and two free-living thiotrophs. This screening showed that ARP-encoding genes are comparatively rare in the related bacteria, but occur in exceptionally high numbers in the *B. thermophilus* symbiont (see Supplementary Discussion IV, Supplementary Table S6a, Supplementary Figs. S1 and S5). While the exact function of ARPs in *Bathymodiolus* thiotrophs is unknown, several possible scenarios are conceivable (see Supplementary Discussion V for details): (a) ARPs might be involved in symbiont colonization of host tissue, because most of them were adhesins, invasins, cadherins, integrins, intimins, and other proteins known to play crucial roles in pathogenic bacteria during host colonization and persistence [41–44]. (b) Their extraordinarily high abundance in *B. thermophilus* may additionally suggest a role in attachment of symbiont cells to each other, i.e., the formation of a biofilm-like structure, or some kind of extracellular proteinaceous matrix around the symbiont cells. This matrix could, for example, serve as proteinaceous substrate that is leaked from the symbionts to the host. As *B. thermophilus* presumably relies relatively more on its symbiont for nutrition than *B. azoricus* (see above), higher abundances of leaked symbiont proteins (e.g., ARPs) might be required. (c) Several of the symbiont ARPs contained domains known to bind and interact with phages (e.g., Ig-like, fibronectin Type 3, immunoglobulin superfamily and C-type lectins [45, 46]), which may indicate that the proposed ARP matrix could protect the symbionts from phages (Supplementary Fig. S4, Supplementary Table S7). Moreover, as previously suggested for pathogens [47, 48], ARPs could enable the symbionts to interact with host phagocytes, potentially enabling them to circumvent host-induced apoptosis (Supplementary Fig. S3). Further in-depth studies will be required to verify these hypotheses.

## Conclusion

Although *B. thermophilus* and *B. azoricus* holobionts are phylogenetically closely related, many of their host–symbiont interactions differ distinctly on the molecular level. Further studies are required to disentangle the respective influence of habitat conditions, biological host parameters (e.g., age, reproductive status), and of individual host–symbiont constellations. However, our results imply that a high degree of variability, even between closely related species, needs to be taken into account when studying host–microbe associations in model systems.

## Supplementary information

Supplementary Online Material

Supplementary Figure S1

Supplementary Figure S2

Supplementary Figure S3

Supplementary Figure S4

Supplementary Figure S5

Supplementary Table S1

Supplementary Table S2

Supplementary Table S3

Supplementary Table S4

Supplementary Table S5

Supplementary Table S6

Supplementary Table S7

Supplementary Table S8
