# Supplementary material for: Comparative proteomics of related symbiotic mussel species reveals high variability of host–symbiont interactions
Source: ISME J. 2019 Nov 4;14(2):649–56. doi: 10.1038/s41396-019-0517-6 (PMC6976577; doi:10.1038/s41396-019-0517-6)
Supplement: Supplementary file 1 — Supplementary Online Material [file 41396_2019_517_MOESM1_ESM.pdf]

Supplementary Online Material

Comparative proteomics of related symbiotic mussel species reveals high variability of host-symbiont interactions

Ruby Ponnudurai[1,2], Stefan E. Heiden[1], Lizbeth Sayavedra[3,4], Tjorven Hinzke[1,5], Manuel Kleiner[6], Christian Hentschker[7], Horst Felbeck[8], Stefan M. Sievert[9], Rabea Schlüter[10], Dörte Becher[5,7], Thomas Schweder[1,5], Stephanie Markert[1,5]

[1]University of Greifswald, Institute of Pharmacy, Greifswald, Germany

[2]European Molecular Biology Laboratory, Meyerhofstrasse 1, 69117 Heidelberg, Germany

[3]Max Planck Institute for Marine Microbiology, Bremen, Germany

[4]Quadram Institute of Bioscience, Norwich, UK

[5]Institute of Marine Biotechnology, Greifswald, Germany

[6]North Carolina State University, Department of Plant & Microbial Biology, Raleigh, NC, USA

[7]University of Greifswald, Institute of Microbiology, Greifswald, Germany

[8]Scripps Institution of Oceanography, La Jolla, CA, USA

[9]Woods Hole Oceanographic Institution, Woods Hole, MA, USA

[10]University of Greifswald, Imaging Center of the Department of Biology, Greifswald, Germany

Correspondence: stephanie.markert@uni-greifswald.de

Contents

Supplementary Methods 3

Supplementary Results and Discussion 15

Supplementary References 22

The following Tables and Figures are provided as separate files:

Supplementary Table S1:	a) Overview of all MS/MS measurements and sample types<br>b) Genomes used for comparative genomics<br>c) Components of the protein database<br>d) Number of protein identifications in all samples
Supplementary Table S2:	a) *B. thermophilus* host and symbiont proteome<br>b) Comparison of *B. thermophilus* sample types (statistics)<br>c) Comparison grouping overview
Supplementary Table S3:	*B. azoricus* host and symbiont proteome
Supplementary Table S4:	a) Comparison *B. thermophilus* vs *B. azoricus* (symbiont fractions)<br>b) Comparison *B. thermophilus* vs *B. azoricus* (gill samples)<br>c) Comparison grouping overview
Supplementary Table S5:	Amino acid biosynthesis in *B. thermophilus* and *B. azoricus*
Supplementary Table S6:	a) Attachment-related genes in four *Bathymodiolus* symbionts<br>b) selected *B. thermophilus* symbiont and host interaction proteins<br>c) selected *B. azoricus* thiotroph and host interaction proteins
Supplementary Table S7:	a) Phage defense-related symbiont proteins in *B. thermophilus*<br>b) Phage defense-related symbiont proteins in *B. azoricus*<br>c) Summed abundance of phage defense-related proteins<br>d) CRISPR repeats in *Bathymodiolus* symbiont genomes<br>e) Phage defense-related genes in four *Bathymodiolus* symbionts
Supplementary Table S8:	a) Spectral counts *B. thermophilus*<br>b) Spectral counts *B. azoricus*<br>c) Biomass contributions *B. thermophilus* and *B. azoricus*<br>d) Spectral identification rates *B. thermophilus* and *B. azoricus*
Supplementary Figure S1:	a) Phylogenetic tree of *B. thermophilus* symbiont integrin<br>b) Phylogenetic tree of *B. thermophilus* symbiont adhesin
Supplementary Figure S2:	Model of CO_2 concentration in *Bathymodiolus*
Supplementary Figure S3:	a) TEM image of *B. thermophilus* gill tissue (single gill filament)<br>b) Lysosomal digestion of symbionts by the host<br>c) Single bacteriocyte (close up)
Supplementary Figure S4:	Phage defense genes in *Bathymodiolus* thiotrophs
Supplementary Figure S5:	BRIG genome comparison of the *B. thermophilus* thiotroph and related bacteria

Supplementary Methods

Sampling of Bathymodiolus mussels

For proteome analyses, three *B. thermophilus* individuals (shell length 105-125 mm) were collected from the Tica vent field on the EPR at 9°50.39′N, 104°17.49′W in 2,511 m water depth on January 13th 2014 during the RV Atlantis cruise AT26-10 by the remotely operated vehicle Jason. Three *B. azoricus* specimens of 60-104 mm shell length were collected from the Menez Gwen vent field on the MAR at 37°50′41″N, 31°31′10″W in 860 m water depth during the RV Meteor cruise M82-3 on September 10th 2010 as described previously (Sayavedra *et al.*, 2015). The bivalves were dissected on board, and gills and foot tissue samples were separately frozen immediately. Fluid temperature at the time of sampling was 3.5°C for the *B. thermophilus* specimens sampled (slightly above the ambient deep seawater temperature of 1.9°C) and 8.9°C for the *B. azoricus* specimens. As vent fluid temperature and sulfide concentrations are usually linearly correlated (Le Bris *et al.*, 2006a; Le Bris *et al.*, 2006b), higher temperatures around the *B. azoricus* specimens at MAR may point to higher sulfide concentrations, compared to the *B. thermophilus* specimens sampled at EPR.

Enrichment of symbiont and host fractions

For enrichment of symbiont and host fractions, gill tissue was homogenized in 1x PBS (Dulbecco's Phosphate Buffered Saline, Sigma-Aldrich). In case of *B. azoricus*, a combination of differential pelleting and rate-zonal density gradient centrifugation was used to a) physically enrich and separate the thiotrophic and methanotrophic symbionts from host bacteriocyte components, and b) to separate the two symbiont types from each other (as described in detail in Ponnudurai *et al.*, 2016). To separate the thiotrophic symbiont fraction from host cell components in *B. thermophilus*, we used differential pelleting by centrifugation (Hinzke *et al.*, 2018; additional density gradient centrifuation was not necessary in this case). Briefly, two batches of 2-3 g gill tissue from a single mussel specimen were homogenized by grating the gill tissue on a metal sieve and then

homogenizing the shreds in a Duall® homogenizer in 2 ml of 1x PBS. The homogenates were then combined and the total volume adjusted with 1x PBS so that gill homogenate to PBS ratio was 1:3 (~15 ml final volume). The homogenate was then slowly centrifuged in a swing-out rotor (5 min, 500 x g, 4 °C) to pellet host nuclei and gill tissue debris. The resultant pellet was subjected to another round of low-speed pelleting and the final pellet was discarded. The supernatant, which contained cytosolic host proteins, bacterial cells and host mitochondria, was transferred to a new 15 ml centrifuge tube and, after brief mixing, distributed into 1.8 ml tubes and centrifuged at maximum speed (20 min, 15.000 x g, 4 °C) in a fixed-angle rotor. The resulting supernatants, which now only contained the enriched cytosolic proteins of the host, were combined into a single 15 ml tube and frozen at -80 °C along with the pellets in the 1.8 ml cryo tubes, which putatively contained the enriched symbiont cells. After each step of this enrichment procedure for *B. thermophilus*, small subsamples (~5 µl) were taken from the gill homogenate, pellets, and host and symbiont enrichments for CARD-FISH analyses, fixed overnight at 4 °C in 1 ml of fixing solution (1-2% paraformaldehyde (PFA) in 1x PBS) and then stored at -80 °C. CARD-FISH subsamples of the *B. azoricus* enrichments were taken as described previously in Ponnudurai *et al.* (2016).

CARD-FISH analyses

PFA-fixed CARD-FISH subsamples of *B. azoricus* and *B. thermophilus* were analyzed under an epifluorescence microscope as described in Ponnudurai *et al.* (2016), based on the technique originally developed by Pernthaler and colleagues (2002), to verify the relative abundance of symbionts and host components in the different enrichments used for proteomic analyses. Briefly, fixed samples were filtered onto GTTP polycarbonate membrane discs with a pore size of 0.2 µm (Millipore, Germany). To visualize the thiotrophic symbionts, the general Gam42a probe tagged with a Cy3 tyramide (Biomers, Germany), along with the unlabeled BET42a as competitor probe (Manz *et al.*, 1992) were hybridized to the thiotrophic symbiont's 23S rRNA on the membrane

discs and amplified. Prior to microscopic evaluation, both the hybridized symbiont cells and host components were counterstained with 1 µg ml^{-1} 4’,6-diamidino-2-phenylindole (DAPI, Sigma-Aldrich). Imaging and counting of signals on the CARD-FISH filters were performed using a Deltavision®RT Image restoration workstation (Applied Precision) with an IX71 microscope (Olympus). Relative abundance of symbiont cells and host cell components in the enriched fractions were estimated by counting the symbiont-specific probe signal against at least 500 DAPI signals per section. DAPI signals which did not overlap with the symbiont probe signals were counted as host cell components (nuclei).

Sample types

The following types of *B. thermophilus* samples were analyzed by proteomics (Supplementary Table S1a): The soluble proteome was extracted from 1) the symbiont-enriched pellet, 2) whole gill tissue and 3) whole foot tissue samples as described previously (Ponnudurai *et al.*, 2016). Digested protein extracts were analyzed using an LTQ-Orbitrap Velos mass spectrometer (Thermo Fisher, Bremen, Germany, see below). In addition, to specifically identify membrane-associated symbiont proteins likely to be involved in host-symbiont interactions, the membrane proteome of whole gill tissue samples was selectively extracted (as described by Eymann *et al.*, 2004) and analyzed in an LTQ-Orbitrap Classic mass spectrometer (Thermo Fisher, Bremen, Germany). All protein analyses were run in three biological replicates (n=3). For *B. azoricus*, we used the existing metaproteomic dataset from Ponnudurai *et al*. (2016), which included the following sample types: 1) The soluble proteome of the symbiont-enriched pellet was analyzed in three biological replicates (n=3) in an LTQ-Orbitrap Classic. Additionally, to increase protein identification rates, two of these three replicates were analyzed as technical replicates in the LTQ-Orbitrap Velos. The soluble proteomes of 2) the enriched host fraction containing host cytosolic proteins, 3) whole gill tissue, and 4) whole foot tissue samples were analyzed in two biological replicates each (n=2) in an LTQ-Orbitrap Velos. The existing *B. azoricus* membrane proteome

data from Ponnudurai *et al*. (2016) obtained from 1) the enriched symbiont fraction and 2) whole gill tissue samples analyzed in an LTQ-Orbitrap Classic were also used to identify symbiont proteins potentially involved in surface-associated processes. In either case, two biological replicates were pooled into one sample for MS analysis.

Protein extraction, LC MS/MS measurements and semi-quantitative data analysis

Extracts of cytosolic proteins and membrane-associated proteins were separated using 1D-PAGE. Liquid chromatography-tandem mass spectrometry (LC-MS/MS) measurements were performed as described previously (Ponnudurai *et al.*, 2016). In brief, an Easy-nLC II (Thermo Fisher Scientific, Waltham, U.S.) was coupled to an LTQ Orbitrap Velos or LTQ Orbitrap Classic mass spectrometer (Thermo Fisher Scientific, MA). Peptide separation was done with a 100 min binary gradient with buffer A (0.1% (v/v) acetic acid) and buffer B (99.9% (v/v) acetonitrile, 0.1% (v/v) acetic acid). The following MS settings were used for LTQ Orbitrap classic measurements: CID fragmentation of the 5 most abundant precursor ions (top5); survey scan resolution: R=30k; exclusion duration: 30 s. During LTQ Orbitrap Velos measurements, the following settings were applied: CID fragmentation of the 20 most abundant precursor ions (top20); survey scan resolution: R=30k; exclusion duration: 20 s.

MS/MS spectra of all *B. thermophilus* and *B. azoricus* samples were searched against an in-house compiled comprehensive target-decoy database containing protein sequences of *Bathymodiolus* symbionts and host (see below). MS results were visualized and filtered using the Scaffold framework (version 4.3.0, http://www.proteomesoftware.com/products/scaffold/) according to the following thresholds: i) 1% peptide false discovery rate (FDR) and 1% protein FDR and ii) at least two unique peptides for each identified protein or protein group. For relative semi-quantitative analysis of the identified proteins, percent normalized spectral abundance factor (%NSAF) values were calculated for each sample (Florens *et al.*, 2006) and for each organism (%OrgNSAFs), i.e., for the respective symbiont(s) and the host (Mueller *et al.*, 2010).

Compilation of the protein database

To enable comparisons between the proteomes of the *B. thermophilus* symbiosis and the *B. azoricus* symbiosis, we used a single database containing sequences targeting all members of both consortia (see Supplementary Table S1c): For identification of host proteins of both *Bathymodiolus* species, amino acid sequences from *B. azoricus* EST libraries (Bettencourt *et al.*, 2010) in the publicly accessible DeepSeaVent database (http://transcriptomics.biocant.pt/deepSeaVent/) were used, since no dedicated genome/transcriptome for *B. thermophilus* exists as yet. *B. thermophilus* symbiont proteins were identified using the recently published *B. thermophilus* symbiont genome (NCBI accession MIQH00000000.1, Ponnudurai *et al.*, 2017). For identification of proteins of the *B. azoricus* thiotroph, we used the protein sequences from the BAZSymA (CDSC00000000.2), BAZSymB (CVUD00000000.2) and the *Bathymodiolus* sp. thiotroph (SouthMAR vent, JGI-IMG genome ID 2518645510) genome assemblies. For the methanotrophic *B. azoricus* symbiont, the *B. azoricus* methanotroph genome assembly (FMJP00000000.1) and the *Bathymodiolus* sp. methanotroph assembly (NCBI BioProject PRJEB13047; Ponnudurai *et al.*, 2016) were used. All sequences were added in a stepwise fashion while simultaneously removing redundant sequences using the CD-Hit-2D clustering program at 100% clustering threshold (Li *et al.*, 2001). Amino acid sequences of common laboratory contaminants were also added to the database. To determine false discovery rates (FDR), the sequences were reversed and appended to the database as decoy sequences.

Calculation of biomass contributions from hosts and symbionts

Spectral counts of all identified proteins were summed up per organism, i.e., for host, thiotrophic symbiont and methanotrophic symbiont in each sample (Supplementary Table S8). The percentage of organism-specific spectral counts relative to all spectral counts in the respective sample corresponds to the respective organisms contribution to proteinaceous biomass in the

sample (Kleiner *et al.*, 2017). Our spectral data analysis pipeline (database search settings and filtering, see above) that produced the spectral count values for biomass calculation was validated using the test data set provided by Kleiner *et al.* (2017). As the *B. thermophilus* host does not have a dedicated genome database, *B. thermophilus* proteins were identified using the *B. azoricus* protein database (see above). Sequence dissimilarities between *B. azoricus* and *B. thermophilus* proteins could potentially cause under-identification of host-derived spectra in *B. thermophilus*, which would lead to an overall reduced number of identified *B. thermophilus* host proteins. However, for gill samples, spectral identification rates, i.e. the ratio of identified spectra to all recorded spectra per sample, were similar for *B. azoricus* and *B. thermophilus*, indicating that even though sequence dissimilarity may lead to loss of identification in *B. thermophilus*, the overall biomass estimates should not be affected (Supplementary Table S8d).

Determination of protein orthologs

To be able to compare symbiont protein abundances between the two *Bathymodiolus* symbioses (Supplementary Figure S4), all thiotrophic symbiont protein sequences identified from gill- and symbiont-enriched fractions of *B. thermophilus* were matched to their respective orthologs identified in gill and gradient pellet fractions of *B. azoricus*. This was done with the Proteinortho tool (Lechner *et al.*, 2011) using an e-value threshold of $1e^{-5}$, 40% sequence coverage and 25% identity of best blast hits. All predicted orthologs were compiled into a single table with their %OrgNSAF values for further comparison using statistics (see below). Proteinortho was also used to identify orthologous attachment-related symbiont proteins (ARPs, Supplementary Table S6a) and phage defense-related symbiont proteins (Supplementary Table S7e) and their respective genes in thiotrophic symbionts of four *Bathymodiolus* host species (*B. thermophilus*, *B. azoricus*, *B. septemdierum* and *Bathymodiolus* sp.).

Statistical testing for significant expression differences between B. azoricus and B. thermophilus protein orthologs

To identify significant differences in symbiont protein abundance between *B. azoricus* and *B. thermophilus* symbiont-enriched samples and in whole gill tissue samples, the protein orthologs table (see above) was filtered as follows: Cross-species identifications were excluded, i.e., *B. thermophilus* symbiont proteins identified based on *B. azoricus* thiotroph sequences were removed, and *B. azoricus* thiotroph proteins identified based on *B. thermophilus* sequences were removed (*B. azoricus* thiotroph proteins identified with sequences of the *Bathymodiolus* sp. thiotroph from SouthMAR vent, which were part of the *B. azoricus database*, were not considered cross-species identifications and were therefore not removed). The following sample groups were compared (see Supplementary Table S4c for an overview): Symbiont-enriched fractions of three *B. thermophilus* individuals (three biological replicates, Orbitrap Velos analysis) were compared against the symbiont-enriched fractions of a) two *B. azoricus* individuals measured by Orbitrap Velos analyses (Group A), b) three *B. azoricus* individuals measured by Orbitrap Classic analyses (Group B), and c) three *B. azoricus* individuals measured by Orbitrap Classic analyses (as biological replicates) and two *B. azoricus* individuals measured by Orbitrap Velos analyses (as technical replicates; Group C). Three biological replicates of *B. thermophilus* whole gill tissue were compared against three biological replicates of *B. azoricus* whole gill tissue (Group D). Expression ratios were averaged across all replicates for each sample type. Host proteins and methanotrophic symbiont proteins were excluded from this analysis. All data tables were loaded into Perseus version 1.5.6.0 (Tyanova and Cox, 2018). Proteins that did not have at least two expression values in at least one group were removed. All remaining values were multiplied by 100. The resulting data matrix was centered-log-ratio- (CLR-) transformed as described in Fernandes *et al*. (2014). Briefly, this involved normalization of each value within a sample against the geometric mean of all values within that sample, followed by log2-transformation of all values within the entire dataset. Missing values produced by the CLR transformation were replaced by a

constant (one-tenth of the smallest value in the entire matrix). This CLR-transformed data matrix containing imputed missing values was then used for statistical testing and calculation of expression ratios. Expression ratios of a protein between two sample types (Figure 1) were obtained by subtraction of corresponding CLR-transformed values. A Welch’s t-test with permutation-based false discovery rate (FDR) of 5% was applied to detect proteins that differed significantly between symbionts of the two host species. The Welch’s test was performed using default settings (sample groupings were preserved for technical replicates during randomizations, both sides, 250 randomizations, s0 parameter: 0). As we aimed to identify major differences between the two thiotrophic *Bathymodiolus* symbionts, the use of three biological replicates was adequate, as it enables the observation of large effect sizes with small variance in a statistically significant manner. By applying a permutation-based false discovery rate of 5%, our statistical testing procedure corrected for the multiple hypothesis testing problem inherent in testing a large number of gene expression differences.

Statistical testing to determine putative symbiosis-specific proteins in B. thermophilus

To identify symbiosis-relevant host and symbiont proteins within *B. thermophilus*, pairwise statistical comparisons of different sample types were performed using Perseus v. 1.5.6.1 (Tyanova and Cox, 2018) with Welch’s t-test and a permutation-based FDR of 0.05 as previously described (Ponnudurai *et al.*, 2016). For an overview of all pairwise comparisons see Supplementary Tables S2b,c. Symbiont proteins that are secreted into the gill bacteriocytes or interact extracellularly with the host are more likely to be detected in whole gill tissue than in enriched symbiont cell fractions. These symbiont proteins with higher abundance in gill tissue than in symbiont-enriched fractions were included in Group A (secreted symbiont proteins). To determine surface-associated symbiont proteins that may be involved in physical interactions or exchange of metabolites with the host, we created a second group (Group B, symbiont cell surface proteins). Group B included i) all symbiont proteins with higher abundance in the gill membrane

proteome than in the soluble proteome of symbiont-enriched fractions, and ii) all symbiont proteins more abundant in the gill membrane proteome than in the soluble gill proteome. To identify symbiosis-related host proteins, all host proteins expressed in higher abundance in symbiont-containing samples (whole gill soluble proteome, whole gill membrane proteome, soluble proteome of symbiont-enriched fraction) than in symbiont-free foot tissue were included in Group C ("symbiosis-specific" host proteins). Finally, we considered host proteins that are potentially involved in direct physical interactions with the symbionts, and that are therefore more abundant in the symbiont-enriched fraction than in whole gill samples as Group D (symbiont-attached host proteins). Statistical analysis of significant differences and calculation of expression ratios was performed using CLR-transformed %OrgNSAF values and the same method as described above.

Assignment of metabolic categories, prediction of protein properties and reconstruction of metabolic pathways

Assignment of COG/KOG metabolic categories, PFAM and TIGRFAM for the identified proteins was done using the online protein annotation pipeline Prophane (http://www.prophane.de/) at default settings. Proteins without a Prophane hit were queried directly against the COG and the PFAM database as described in Ponnudurai *et al.* (2017). Annotations were further consolidated by blasting protein sequences against the NCBI (nr) and UniProt databases. Proteins that had sparse hits in NCBI or were "hypothetical" in function were searched for structural homology using the HHpred server for remote protein homology detection using Hidden-Markov Models (Soding *et al.*, 2005). Transmembrane helices were predicted using the TMHMM Server v. 2.0 (Krogh *et al.*, 2001). For prediction of signal peptides, the SignalP 4.1 Server (Petersen *et al.*, 2011b), PECAS (Cortazar *et al.*, 2015) and Phobius (Kall *et al.*, 2007) were used. Non-classically secreted proteins were identified using the SecretomeP online server (Bendtsen *et al.*, 2005; http://www.cbs.dtu.dk/services/SecretomeP/). The PsortB online server (http://www.psort.org/psortb/) was used to predict the subcellular localization of each symbiont

protein (Yu *et al.*, 2010). Functions of hypothetical proteins were inferred using structural homology searches, such as Phyre (Kelley *et al.*, 2015), and based on their genomic context using the RAST SEED viewer 2.0 (http:// rast.nmpdr.org/, Aziz *et al.*, 2008; Overbeek *et al.*, 2014) and the Artemis genome visualization tool (Carver *et al.*, 2012). Using our protein expression data and the available genome assemblies, metabolic pathways were reconstructed by referring to the KEGG and MetaCyc pathway databases (Caspi *et al.*, 2016).

Genome comparisons

For an overview of all genomes used in our comparative genome analysis see Supplementary Table S1b. Genome sequences of four thiotrophic *Bathymodiolus* symbionts (*B. thermophilus* thioautotrophic gill symbiont strain BAT/CrabSpa’14, acc. no. MIQH01; *B. azoricus* thioautotrophic gill symbiont strains BazSymA and BazSymB, acc. nos. CDSC02 and CVUD02; endosymbiont of *B. septemdierum* strain Myojin Knoll, acc. no. AP013042), of two thiotrophic giant clam symbionts („*Candidatus* Ruthia magnifica“ strain Cm, acc. no. CP000488; „*Candidatus* Vesicomyosocius okutanii“ strain HA, acc. no. AP009247), and of two free-living relatives („*Candidatus* Thioglobus autotrophicus“ strain EF1, acc. no. CP010552; „*Candidatus* Thioglobus singularis“ isolate GG2, acc. no. CP008725) were downloaded from GenBank. An additional genome sequence of a thiotrophic *Bathymodiolus* symbiont was obtained from IMG (*Bathymodiolus* sp. South MAR chemoautotrophic symbiont, IMG Taxon ID 2518645510). Several scripts from the bac-genomics-scripts toolbox (Leimbach, 2016) were used to create artificially concatenated files of incomplete genomes („cat_seq.pl“) for the BLASTN analysis (version 2.7.1+, „-task blastn -evalue 2e-10 -dust no“; Altschul *et al.*, 1990; Camacho *et al.*, 2009) and for finding regions of difference (RODs) between the genomes („blast_rod_finder.pl“). The ROD threshold („-m 2400“) was set slightly smaller than the average contig size (3,088,407 bp/1,281 ≈ 2,410.93 bp). The results were then visualized with the BLAST Ring Image Generator (BRIG; Alikhan *et al.*, 2011).

Determination of attachment-related genes and CRISPR repeats in Bathymodiolus symbionts

Nucleotide sequences of the thiotrophic symbionts from *B. thermophilus*, *B. azoricus* (BAZSymA and BAZSymB), *B. septemdierum* and *Bathymodiolus* sp. (see Supplementary Table S1b for details) were uploaded to the eggNOG mapper server at http://eggnogdb.embl.de/#/app/emapper (Huerta-Cepas *et al.*, 2016) and run in DIAMOND mode using default settings. All symbiont sequences were scanned for conserved domains using rps-BLAST against the Conserved Domains Database (CDD; ftp://ftp.ncbi.nih.gov/pub/mmdb/cdd; e-value: 0.01; Marchler-Bauer *et al.*, 2010). For *B. thermophilus* and *B. azoricus* symbiont sequences, the functional annotations obtained from Prophane (see above) were also included. Genes were considered attachment- or toxin-related, if at least one of the annotation pipelines predicted a respective function (based on a text search for the keywords "virulence", "cadherin", "adhesion", "integrin", "lectin", "binding", "toxin", "RTX", "Rhs", "Ig-like", and "TPR") in any of the gene orthologs (see Supplementary Table S6a). All orthologs of the same gene in the other symbiont genomes were then automatically assigned the same function. CRISPR repeats in the *B. thermophilus* symbiont and the thiotrophic *B. azoricus* symbiont were identified by scanning the respective genomes using the CRISPRfinder tool (Grissa *et al.*, 2007).

Phylogenetic analysis of symbiont adhesins and integrins

To reconstruct the phylogenies of integrin and adhesion genes, we used the sequences of the *B. thermophilus* thiotroph, which showed highest abundance on the protein level, as query to obtain related sequences from the Refseq protein data base (e.g. OJA03427 for adhesins and OIR23680 for integrins). Sequences were aligned with MAFFT (Katoh *et al.*, 2002). The alignment was masked to use only those positions that were present in at least 75% of the sequences using Geneious V9 (http://www.geneious.com; Kearse *et al.*, 2012), which resulted in an alignment of 1,037 amino acid positions for integrins and 3,604 amino acids for adhesins. Maximum-likelihood phylogenetic reconstructions were done with RAxML (Stamatakis, 2014) with bootstrap support

calculated from 100 replicates. The phylogenetic trees (Supplementary Figures S1a,b) were edited with iTOL (Letunic and Bork, 2016).

Electron microscopy

B. thermophilus gill tissue for electron microscopy was sampled during Atlantis cruise AT37-12 to the EPR in 2017. After recovery, the animals were stored at 4 °C in seawater before dissection (no longer than 4 h). Individual gill tissue sections were separately fixed in fixative containing 4% paraformaldehyde, 50 mM HEPES, 10% sucrose and 1% glutaraldehyde (GA, added directly before use) for 1-1.5 h at room temperature. Samples were then stored at 4 °C until further processing. Fixed samples were washed three times with washing buffer (100 mM cacodylate buffer [pH 7.0], 1 mM calcium chloride, 0.09 M sucrose) for 10 min each step and treated with 1% osmium tetroxide in washing buffer for 1 h at room temperature. After washing, samples were dehydrated in a graded series of ethanol (30%, 50%, 70%, 90%, and 100%) on ice for 30 min each step. Afterwards, the material was infiltrated with the acrylic resin LR White in a stepwise fashion as described by Hammerschmidt *et al.* (2005). Sections were cut with a diamond knife on an ultramicrotome (Reichert Ultracut, Leica UK Ltd, Milton Keynes, UK), stained with 4% aqueous uranyl acetate for 5 min and finally examined with a transmission electron microscope LEO 906 (Carl Zeiss Microscopy GmbH, Oberkochen, Germany) at an acceleration voltage of 80 kV. All micrographs were edited using Adobe Photoshop CS6.

Supplementary Results and Discussion

I. Total protein identifications and biomass contributions

Our in-depth proteomic analyses of the thiotrophic *B. thermophilus* symbiosis from the East Pacific Rise (EPR) yielded a total of 3,474 protein identifications (thiotrophic symbiont: 1,523 proteins, host: 1,951 proteins; Supplementary Table S2a). For the dual *B. azoricus* symbiosis from the Mid-Atlantic Ridge (MAR) we identified 4,572 proteins (thiotrophic symbiont: 1,154, methanotrophic symbiont: 441, host: 2,977; Supplementary Table S3; for an overview of protein identification numbers in all sample types analyzed in this study see Supplementary Table S1d). Based on these host and symbiont spectral identifications, we calculated the relative proportion of symbiont biomass in both hosts and found that *B. thermophilus* harbored a notably larger symbiont population than *B. azoricus* (see main text, Figure 2, Supplementary Table S8). Although a previous study estimated symbiont biomass per gram wet weight in *B. azoricus* gills to be similar to symbiont biomass in *B. thermophilus* (Martins *et al.*, 2008), this is not necessarily in disagreement with our results, but could be attributed to dissimilar biomass assessment methods.

II. Expression of carbonic anhydrase differs greatly between the two host species

We detected the host enzyme carbonic anhydrase (CA) in approximately 100-fold higher abundance in *B. azoricus* samples compared to *B. thermophilus* samples (main text). In the absence of a dedicated *B. thermophilus* host genome sequence, all *B. thermophilus* host proteins had to be identified using *B. azoricus* host EST sequences. As eukaryotic CAs can be quite diverse (Le Roy *et al.*, 2014), we cannot exclude the possibility that an unknown *B. thermophilus*-specific CA may have escaped identification. It seems, however, unlikely that this could account for the enormous difference observed in CA abundances between *B. azoricus* and *B. thermophilus*, because 1) not just one, but all *B. thermophilus* CAs would have to be completely

different from those in *B. azoricus*. 2) Even if their overall sequences differ, proteins of the same function can be expected to share at least some of their peptides and can thus be identified, particularly if they are highly abundant. 3) Overall identification rates of *B. thermophilus* host proteins in our data set were not notably lower than those for *B. azoricus*. Therefore, database specificity seems to play a minor role in the observed differences in CA abundance. Instead, we suggest that in *B. azoricus*, CA could "trap" methanotroph-derived CO_2 by conversion into the non-diffusible HCO_3^-. This would create a concentrated but immobilized pool of CO_2, which can be successively released for carbon fixation by the thiotrophic symbionts in *B. azoricus* (see main text). It remains to be elucidated in future studies how the trapped bicarbonate can be shuttled between the two symbionts in *B. azoricus*. CA abundance in *B. azoricus* and *B. thermophilus* might also be regulated in response to other factors, such as external CO_2 concentrations, sulfide availability and symbiont carbon fixation rates (Scott, 2003; Scott and Cavanaugh, 2007).

III. The provision of host intermediates may allow for symbiont population control

In *Bathymodiolus*, the CO_2 reservoir established by carbonic anhydrase (see main text and above) could not only fuel bacterial CO_2 fixation, but may also support the generation of oxaloacetate by the host enzymes phosphoenolpyruvate carboxykinase and pyruvate carboxyltransferase (Supplementary Figure S2). Thiotrophic *Bathymodiolus* symbionts seem to lack the genetic potential to replenish their oxaloacetate pool autonomously (Ponnudurai *et al.*, 2016, this study), and likely rely on their hosts to compensate for this deficiency. This may have important implications for the suggested cycling of amino acids between *Bathymodiolus* symbionts and hosts (Figure 3B, main text): The transamination of host glutamate in the symbiont requires the presence of oxaloacetate as the amino group donor in the symbiont cell. In light of the *Bathymodiolus* thiotrophs' apparent inability to produce oxaloacetate, this suggests that the host needs to provide both, glutamate and oxaloacetate in order to receive amino acids from its symbiont. This would enable the host not only to directly control the symbiont's amino acid

metabolism, but also to exert population control by supporting the symbiont with essential dicarboxylates.

IV. Symbiont attachment-related proteins (ARPs) – genome comparison and phylogenetic analysis

With 22, 3 and 10 ARP-encoding genes, the genomes of the *B. azoricus*, *B. septemdierum* and *Bathymodiolus* sp. thiotrophs, respectively, contain a much smaller attachment-related protein repertoire than the *B. thermophilus* symbiont genome (266 genes). Moreover, many of the *B. thermophilus* symbiont’s ARPs are encoded in genome regions, which have no homologs in any of the related *Bathymodiolus* symbiont genomes, nor in the genomes of two thiotrophic clam symbionts or two free-living thiotrophs (“regions of difference” = RODs in Supplementary Figure S5). This finding is supported by our phylogenetic analysis of two *B. thermophilus* symbiont ARPs, an FG-GAP domain-containing adhesin and an integrin (containing an integrin Alpha domain). Both, the integrin and the adhesin, showed very few homologs in related *Bathymodiolus* symbionts (Supplementary Figure S1). The *B. thermophilus* symbiont’s adhesins clustered almost exclusively with each other and the whole group displayed highest similarity to proteins of the free-living *Chlorobium limicola* and *Aureimonas frigidaquae*. Likewise, most *B. thermophilus* symbiont integrins showed highest similarity to each other, but not to integrins of related bacteria, suggesting that these genes are the result of multiple gene duplication events and may originally have been acquired by horizontal gene transfer. The most closely related proteins from other organisms were integrins of other *Bathymodiolus* thiotrophs and of free-living cyanobacteria and Rhodobacterales.

V. Possible functions of Bathymodiolus symbiont ARPs

Attachment-related proteins were particularly abundant in the thiotrophic *B. thermophilus* symbiont, and may have several possible functions, which are discussed below (see also main text).

a) Colonization of host tissue

In the gill filaments of adult *Bathymodiolus* mussels, new symbionts are constantly taken up into growing, uncolonized tissue (Won *et al.*, 2003; Wentrup *et al.*, 2014). However, although the gill tissues analyzed in our study included actively growing regions, it seems questionable whether the entire ARP abundance we observed could solely be attributed to symbiont colonization of these very small host tissue areas. Also, this would not explain the immense differences we observed in symbiont ARP abundance between *B. thermophilus* and *B. azoricus*. More likely, ARPs may perform additional essential functions in colonized gill tissue.

b) Nutrient transfer

We detected a multitude of digestive host proteins, i.e. proteases, peptidases, and carbohydrate degradation enzymes in both *Bathymodiolus* hosts (Supplementary Table S6b and c). In *B. thermophilus*, total abundance of these digestive host proteins was substantially higher in gill samples compared to symbiont-free foot samples, indicating that these proteins may be involved in digestion of symbionts or symbiont-derived nutrients (see Supplementary Figure S3 for a TEM image of symbiont digestion). In *B. azoricus*, on the other hand, total digestive protein abundance was only slightly higher in gill tissue, compared to foot tissue, which may indicate that many of these proteins are not specifically involved in digestion of symbiont-derived substrates. This is in accordance with the assumption that *B. thermophilus* may rely relatively more on its symbionts for nutrition, while *B. azoricus* may retrieve a larger part of its diet from filter-feeding (see main text). It might be speculated that highly abundant *B. thermophilus* symbiont ARPs present a

proteinaceous substrate that is secreted by the symbiont and digested by the host. However, as total abundances of protein degradation-specific host proteins were almost equal in *B. thermophilus* (2.46 %OrgNSAF) and *B. azoricus* gills (2.41 %OrgNSAF), this hypothesis will require further investigations.

c) Protection from phages or from host immunity and apoptosis

We detected various phage defense-related proteins and mobile genetic elements of the CRISPR-Cas and restriction-modification (R-M) systems in the *Bathymodiolus* thiotrophs (Supplementary Table S7, Supplementary Figure S4), implying that exposure to phages is common in these symbioses. Phages are abundant in diffuse-flow vent fluids (Ortmann and Suttle, 2005) and phage infections can easily be detrimental to the entire symbiont population in a bacterial "monoculture" such as the *Bathymodiolus* symbiosis. Considering that many of the symbiont ARPs (9 %OrgNSAF in *B. thermophilus* gill tissue, Supplementary Table S6b) contain domains that have been implicated in virus interactions (Ig-like, fibronectin Type 3, immunoglobulin superfamily and C-type lectins Fraser *et al.*, 2006; Barr *et al.*, 2013), a putative extracellular ARP matrix may therefore function as a barrier that prevents phage intrusion. Supporting this idea of a protective proteinaceous biofilm, Ig-like proteins, adhesins and other attachment-related proteins have been associated to biofilm formation in pathogens (De Gregorio et al., 2015; Fong and Yildiz, 2015).

ARPs in thiotrophic *Bathymodiolus* symbionts may furthermore play a yet to be determined role in assisting the bacteria to evade host immune cells, apoptotic factors or digestion by phagocytosis (Supplementary Figure S3), enabling the symbionts to stably persist within the host tissue. We identified various immune-related host proteins (carbohydrate-binding proteins, lectins, and immunoglobulins), and apoptotic factors (mostly caspases, Supplementary Table S6b and c). Many of them were enriched in symbiont-containing tissue compared to foot tissue, indicating that these host immunity proteins are involved in interactions with (or against) the

symbionts. The *Bathymodiolus* innate immune system is known to exhibit phagocytic activities (Bettencourt *et al.*, 2010; Martins *et al.*, 2014; Tame *et al.*, 2015), and apoptotic factors were supposed to play crucial roles in inducing symbiont death in a putative host-induced symbiont population control mechanism (Guezi *et al.*, 2013; Wong *et al.*, 2015; Sun *et al.*, 2017; Zheng *et al.*, 2017; Piquet *et al.*, 2019). The symbionts may therefore have developed strategies to counteract or avoid this potential threat. In pathogens, ARPs were shown to interact with phagocytes of infected host cells, enabling the bacteria to circumvent the host’s innate immune response (Baorto *et al.*, 1997; Pizarro-Cerdá and Cossart, 2006). In the squid symbiont *Vibrio fischeri*, a hitherto unknown signal that is secreted through an outer membrane porin prevents adhesion of symbionts to host hemocytes, thus allowing the bacteria to escape phagocytosis (Nyholm *et al.*, 2009). The *B. thermophilus* symbiont ARPs might have a similar signal function. As previously suggested for toxin-like proteins in *Bathymodiolus* symbionts (Sayavedra *et al.*, 2015), ARPs in *Bathymodiolus* thiotrophs may thus represent “tamed”, beneficial versions of their pathogenic counterparts that play crucial roles in host-microbe interactions.

We hypothesize that *Bathymodiolus* symbionts may secrete ARPs through their outer membrane-associated porins (Omps), which show high structural homology to the above mentioned *V. fischeri* porin OmpU (Omp1: 98.5% structural homology, 15% sequence similarity; Omp2: 97.5% structural homology, 14% sequence similarity). Omp1 and Omp2 were furthermore extraordinarily abundant in the *B. thermophilus* symbiont (compared to the *B. azoricus* thiotroph, Figure 1), possibly because the high abundance of ARP effector proteins in this symbiont necessitates high abundance of pores for ARP secretion.

Further in-depth studies will be required to test these hypotheses. These follow-up studies could include heterologous overexpression of individual ARPs, which would provide the basis for functional assays, as well as for structural biological analyses, which may identify specific functional domains. Moreover, purified enrichments of these heterologously expressed proteins

could be tested for their ability to form complexes with host proteins (e.g. in host tissue homogenate) in pull-down assays, and specific antibodies raised against selected regions of heterologously expressed ARPs could allow localization of these proteins in host tissue or even in individual host cell compartments using electron microscopy.

Supplementary References

Alikhan N-F, Petty NK, Ben Zakour NL, Beatson SA. (2011). BLAST Ring Image Generator (BRIG): simple prokaryote genome comparisons. *BMC Genomics* **12:** 402.

Altschul SF, Gish W, Miller W, Myers EW, Lipman DJ. (1990). Basic local alignment search tool. *J Mol Biol* **215:** 403-410.

Aziz RK, Bartels D, Best AA, DeJongh M, Disz T, Edwards RA *et al*. (2008). The RAST Server: rapid annotations using subsystems technology. *BMC Genomics* **9:** 75.

Baorto DM, Gao Z, Malaviya R, Dustin ML, van der Merwe A, Lublin DM *et al*. (1997). Survival of FimH-expressing enterobacteria in macrophages relies on glycolipid traffic. *Nature* **389:** 636–639.

Barr JJ, Auro R, Furlan M, Whiteson KL, Erb ML, Pogliano J *et al*. (2013). Bacteriophage adhering to mucus provide a non-host-derived immunity. *Proc Natl Acad Sci U S A* **110:** 10771-10776.

Bendtsen JD, Kiemer L, Fausboll A, Brunak S. (2005). Non-classical protein secretion in bacteria. *BMC Microbiol* **5:** 58.

Bettencourt R, Pinheiro M, Egas C, Gomes P, Afonso M, Shank T *et al*. (2010). High-throughput sequencing and analysis of the gill tissue transcriptome from the deep-sea hydrothermal vent mussel *Bathymodiolus azoricus*. *BMC Genomics* **11:** 559-559.

Camacho C, Coulouris G, Avagyan V, Ma N, Papadopoulos J, Bealer K *et al*. (2009). BLAST+: architecture and applications. *BMC Bioinformatics* **10:** 421.

Carver T, Harris SR, Berriman M, Parkhill J, McQuillan JA. (2012). Artemis: an integrated platform for visualization and analysis of high-throughput sequence-based experimental data. *Bioinformatics* **28:** 464-469.

Caspi R, Billington R, Ferrer L, Foerster H, Fulcher CA, Keseler IM *et al*. (2016). The MetaCyc database of metabolic pathways and enzymes and the BioCyc collection of pathway/genome databases. *Nucleic Acids Res* **44:** D471-D480.

Cortazar AR, Oguiza JA, Aransay AM, Lavin JL. (2015). PECAS: prokaryotic and eukaryotic classical analysis of secretome. *Amino Acids* **47:** 2659-2663.

Eymann C, Dreisbach A, Albrecht D, Bernhardt J, Becher D, Gentner S *et al*. (2004). A comprehensive proteome map of growing *Bacillus subtilis* cells. *PROTEOMICS* **4:** 2849-2876.

Fernandes AD, Reid JN, Macklaim JM, McMurrough TA, Edgell DR, Gloor GB. (2014). Unifying the analysis of high-throughput sequencing datasets: characterizing RNA-seq, 16S rRNA gene sequencing and selective growth experiments by compositional data analysis. *Microbiome* **2:** 15.

Fiala-Médioni A, Métivier C, Herry A, Le Pennec M. (1986). Ultrastructure of the gill of the hydrothermal-vent mytilid *Bathymodiolus* sp. *Mar Biol* **92:** 65-72.

Florens L, Carozza MJ, Swanson SK, Fournier M, Coleman MK, Workman JL *et al*. (2006). Analyzing chromatin remodeling complexes using shotgun proteomics and normalized spectral abundance factors. *Methods* **40:** 303-311.

Fraser JS, Yu Z, Maxwell KL, Davidson AR. (2006). Ig-like domains on bacteriophages: a tale of promiscuity and deceit. *J Mol Biol* **359:** 496-507.

Grissa I, Vergnaud G, Pourcel C. (2007). CRISPRFinder: a web tool to identify clustered regularly interspaced short palindromic repeats. *Nucleic Acids Res* **35:** W52-W57.

Guezi H, Boutet I, Tanguy A, Lallier F. (2013). The potential implication of apoptosis in the control of chemosynthetic symbionts in the control of chemosynthetic symbionts in *Bathymodiolus thermophilus*. *Fish Shellfish Immunol* **34:** 1709.

Hammerschmidt S, Wolff S, Hocke A, Rosseau S, Müller E, Rohde M. (2005). Illustration of Pneumococcal Polysaccharide Capsule during Adherence and Invasion of Epithelial Cells. *Infect Immun* **73:** 4653-4667.

Hinzke T, Kleiner M, Markert S (2018). Centrifugation-Based Enrichment of Bacterial Cell Populations for Metaproteomic Studies on Bacteria–Invertebrate Symbioses. In: Becher D (ed). *Microbial Proteomics: Methods and Protocols*. Springer New York: New York, NY. pp 319-334.

Huerta-Cepas J, Szklarczyk D, Forslund K, Cook H, Heller D, Walter MC *et al*. (2016). eggNOG 4.5: a hierarchical orthology framework with improved functional annotations for eukaryotic, prokaryotic and viral sequences. *Nucleic Acids Res* **44:** D286-D293.

Ikuta T, Takaki Y, Nagai Y, Shimamura S, Tsuda M, Kawagucci S *et al*. (2015). Heterogeneous composition of key metabolic gene clusters in a vent mussel symbiont population. *ISME J* **10:** 990-1001.

Kall L, Krogh A, Sonnhammer EL. (2007). Advantages of combined transmembrane topology and signal peptide prediction--the Phobius web server. *Nucleic Acids Res* **35:** W429-432.

Katoh K, Misawa K, Kuma Ki, Miyata T. (2002). MAFFT: a novel method for rapid multiple sequence alignment based on fast Fourier transform. *Nucleic Acids Res* **30:** 3059-3066.

Kearse M, Moir R, Wilson A, Stones-Havas S, Cheung M, Sturrock S *et al*. (2012). Geneious Basic: An integrated and extendable desktop software platform for the organization and analysis of sequence data. *Bioinformatics* **28:** 1647-1649.

Kelley LA, Mezulis S, Yates CM, Wass MN, Sternberg MJE. (2015). The Phyre2 web portal for protein modeling, prediction and analysis. *Nat Protocols* **10:** 845-858.

Kleiner M, Thorson E, Sharp CE, Dong X, Liu D, Li C *et al*. (2017). Assessing species biomass contributions in microbial communities via metaproteomics. *Nat Commun*. **8**, 1558, DOI: 10.1038/s41467-017-01544-x.

Krogh A, Larsson B, von Heijne G, Sonnhammer EL. (2001). Predicting transmembrane protein topology with a hidden Markov model: application to complete genomes. *J Mol Biol* **305:** 567-580.

Kuwahara H, Takaki Y, Yoshida T, Shimamura S, Takishita K, Reimer JD *et al*. (2008). Reductive genome evolution in chemoautotrophic intracellular symbionts of deep-sea *Calyptogena* clams. *Extremophiles* **12:** 365-374.

Le Bris N, Govenar B, Le Gall C, Fisher CR. (2006a). Variability of physico-chemical conditions in 9°50'N EPR diffuse flow vent habitats. *Mar Chem* **98:** 167-182.

Le Bris N, Rodier P, Sarradin P-M, Le Gall C. (2006b). Is temperature a good proxy for sulfide in hydrothermal vent habitats? *Cah Biol Mar* **47:** 465-470.

Le Roy N, Jackson DJ, Marie B, Ramos-Silva P, Marin F. (2014). The evolution of metazoan α-carbonic anhydrases and their roles in calcium carbonate biomineralization. *Front Zool* **11:** 75.

Lechner M, Findeiß S, Steiner L, Marz M, Stadler PF, Prohaska SJ. (2011). Proteinortho: Detection of (Co-) orthologs in large-scale analysis. *BMC Bioinformatics* **12:** 124.

Leimbach A. (2016). bac-genomics-scripts: Bovine *E. coli* mastitis comparative genomics edition. *Zenodo*. DOI: http://dx.doi.org/10.5281/zenodo.215824.

Letunic I, Bork P. (2016). Interactive tree of life (iTOL) v3: an online tool for the display and annotation of phylogenetic and other trees. *Nucleic Acids Res* **44:** W242-W245.

Li W, Jaroszewski L, Godzik A. (2001). Clustering of highly homologous sequences to reduce the size of large protein databases. *Bioinformatics* **17:** 282-283.

Manz W, Amann R, Ludwig W, Wagner M, Schleifer K-H. (1992). Phylogenetic Oligodeoxynucleotide Probes for the Major Subclasses of Proteobacteria: Problems and Solutions. *Syst Appl Microbiol* **15:** 593-600.

Marchler-Bauer A, Lu S, Anderson JB, Chitsaz F, Derbyshire MK, DeWeese-Scott C *et al*. (2010). CDD: a Conserved Domain Database for the functional annotation of proteins. *Nucleic Acids Res* **39:** D225-D229.

Marshall KT, Morris RM. (2015). Genome Sequence of "*Candidatus* Thioglobus singularis" Strain PS1, a Mixotroph from the SUP05 Clade of Marine Gammaproteobacteria. *Genome Announc* **3:** e01155-01115.

Martins E, Figueras A, Novoa B, Santos RS, Moreira R, Bettencourt R. (2014). Comparative study of immune responses in the deep-sea hydrothermal vent mussel *Bathymodiolus azoricus* and the shallow-water mussel *Mytilus galloprovincialis* challenged with *Vibrio* bacteria. *Fish Shellfish Immunol* **40:** 485-499.

Martins I, Colaço A, Dando PR, Martins I, Desbruyères D, Sarradin P-M *et al*. (2008). Size-dependent variations on the nutritional pathway of *Bathymodiolus azoricus* demonstrated by a C-flux model. *Ecol Modell* **217:** 59-71.

Moran NA, McCutcheon JP, Nakabachi A. (2008). Genomics and evolution of heritable bacterial symbionts. *Annu Rev Genet* **42:** 165-190.

Mueller RS, Denef VJ, Kalnejais LH, Suttle KB, Thomas BC, Wilmes P *et al*. (2010). Ecological distribution and population physiology defined by proteomics in a natural microbial community. *Mol Syst Biol*. **6**, 374, DOI: 10.1038/Msb.2010.30.

Newton IL, Girguis PR, Cavanaugh CM. (2008). Comparative genomics of vesicomyid clam (Bivalvia: Mollusca) chemosynthetic symbionts. *BMC Genomics* **9:** 585.

Nyholm SV, Stewart JJ, Ruby EG, McFall-Ngai MJ. (2009). Recognition between symbiotic *Vibrio fischeri* and the haemocytes of *Euprymna scolopes*. *Environ Microbiol* **11:** 483-493.

Ortmann AC, Suttle CA. (2005). High abundances of viruses in a deep-sea hydrothermal vent system indicates viral mediated microbial mortality. *Deep Sea Res Part 1 Oceanogr Res Pap* **52:** 1515-1527.

Overbeek R, Olson R, Pusch GD, Olsen GJ, Davis JJ, Disz T *et al*. (2014). The SEED and the Rapid Annotation of microbial genomes using Subsystems Technology (RAST). *Nucleic Acids Res* **42:** D206-214.

Pernthaler A, Pernthaler J, Amann R. (2002). Fluorescence in situ hybridization and catalyzed reporter deposition for the identification of marine bacteria. *Appl Environ Microbiol* **68:** 3094-3101.

Petersen JM, Zielinski FU, Pape T, Seifert R, Moraru C, Amann R *et al*. (2011a). Hydrogen is an energy source for hydrothermal vent symbioses. *Nature* **476:** 176-180.

Petersen TN, Brunak S, von Heijne G, Nielsen H. (2011b). SignalP 4.0: discriminating signal peptides from transmembrane regions. *Nat Methods* **8:** 785-786.

Piquet B, Shillito B, Lallier FH, Duperron S, Andersen AC. (2019). High rates of apoptosis visualized in the symbiont-bearing gills of deep-sea *Bathymodiolus* mussels. *PloS one* **14:** e0211499.

Pizarro-Cerdá J, Cossart P. (2006). Bacterial Adhesion and Entry into Host Cells. *Cell* **124:** 715-727.

Ponnudurai R, Kleiner M, Sayavedra L, Petersen JM, Moche M, Otto A *et al*. (2016). Metabolic and physiological interdependencies in the *Bathymodiolus azoricus* symbiosis. *ISME J* **11:** 463-477.

Ponnudurai R, Sayavedra L, Kleiner M, Heiden SE, Thurmer A, Felbeck H *et al*. (2017). Genome sequence of the sulfur-oxidizing *Bathymodiolus thermophilus* gill endosymbiont. *Stand Genomic Sci* **12:** 50.

Roeselers G, Newton ILG, Woyke T, Auchtung TA, Dilly GF, Dutton RJ *et al*. (2010). Complete genome sequence of *Candidatus* Ruthia magnifica. *Stand Genomic Sci* **3:** 163-173.

Sayavedra L, Kleiner M, Ponnudurai R, Wetzel S, Pelletier E, Barbe V *et al*. (2015). Abundant toxin-related genes in the genomes of beneficial symbionts from deep-sea hydrothermal vent mussels. *eLife*. **4**, e07966, DOI: 10.7554/eLife.07966.

Scott KM. (2003). A delta13C-based carbon flux model for the hydrothermal vent chemoautotrophic symbiosis *Riftia pachyptila* predicts sizeable CO(2) gradients at the host-symbiont interface. *Environ Microbiol* **5:** 424-432.

Scott KM, Cavanaugh CM. (2007). CO_2 uptake and fixation by endosymbiotic chemoautotrophs from the bivalve *Solemya velum*. *Appl Environ Microbiol* **73:** 1174–1179.

Shah V, Morris RM. (2015). Genome Sequence of "*Candidatus* Thioglobus autotrophica" Strain EF1, a Chemoautotroph from the SUP05 Clade of Marine Gammaproteobacteria. *Genome Announc* **3:** e01156-01115.

Soding J, Biegert A, Lupas AN. (2005). The HHpred interactive server for protein homology detection and structure prediction. *Nucleic Acids Res* **33:** W244-248.

Stamatakis A. (2014). RAxML version 8: a tool for phylogenetic analysis and post-analysis of large phylogenies. *Bioinformatics* **30:** 1312-1313.

Sun J, Zhang Y, Xu T, Zhang Y, Mu H, Zhang Y *et al*. (2017). Adaptation to deep-sea chemosynthetic environments as revealed by mussel genomes. *Nat Ecol Evol*. **1**, 0121, DOI: 10.1038/s41559-017-0121.

Tame A, Yoshida T, Ohishi K, Maruyama T. (2015). Phagocytic activities of hemocytes from the deep-sea symbiotic mussels *Bathymodiolus japonicus*, *B. platifrons*, and *B. septemdierum*. *Fish Shellfish Immunol* **45:** 146-156.

Tyanova S, Cox J (2018). Perseus: A Bioinformatics Platform for Integrative Analysis of Proteomics Data in Cancer Research. In: von Stechow L (ed). *Cancer Systems Biology*. Humana Press: New York. pp 133-148.

Wentrup C, Wendeberg A, Schimak M, Borowski C, Dubilier N. (2014). Forever competent: deep-sea bivalves are colonized by their chemosynthetic symbionts throughout their lifetime. *Environ Microbiol* **16:** 3699-3713.

Won YJ, Hallam SJ, O'Mullan GD, Pan IL, Buck KR, Vrijenhoek RC. (2003). Environmental acquisition of thiotrophic endosymbionts by deep-sea mussels of the genus *Bathymodiolus*. *Appl Environ Microbiol* **69:** 6785-6792.

Wong YH, Sun J, He LS, Chen LG, Qiu JW, Qian PY. (2015). High-throughput transcriptome sequencing of the cold seep mussel *Bathymodiolus platifrons*. *Sci Rep* **5:** 16597.

Yu NY, Wagner JR, Laird MR, Melli G, Rey S, Lo R *et al*. (2010). PSORTb 3.0: improved protein subcellular localization prediction with refined localization subcategories and predictive capabilities for all prokaryotes. *Bioinformatics* **26:** 1608-1615.

Zheng P, Wang M, Li C, Sun X, Wang X, Sun Y *et al*. (2017). Insights into deep-sea adaptations and host–symbiont interactions: A comparative transcriptome study on *Bathymodiolus* mussels and their coastal relatives. *Mol Ecol* **26:** 5133-5148.
