# Supplementary material for: Comparative proteomics of related symbiotic mussel species reveals high variability of host–symbiont interactions
Source: ISME J. 2019 Nov 4;14(2):649–56. doi: 10.1038/s41396-019-0517-6 (PMC6976577; doi:10.1038/s41396-019-0517-6)
Supplement: Supplementary file 2 — Supplementary Figure S1 [file 41396_2019_517_MOESM2_ESM.pdf]

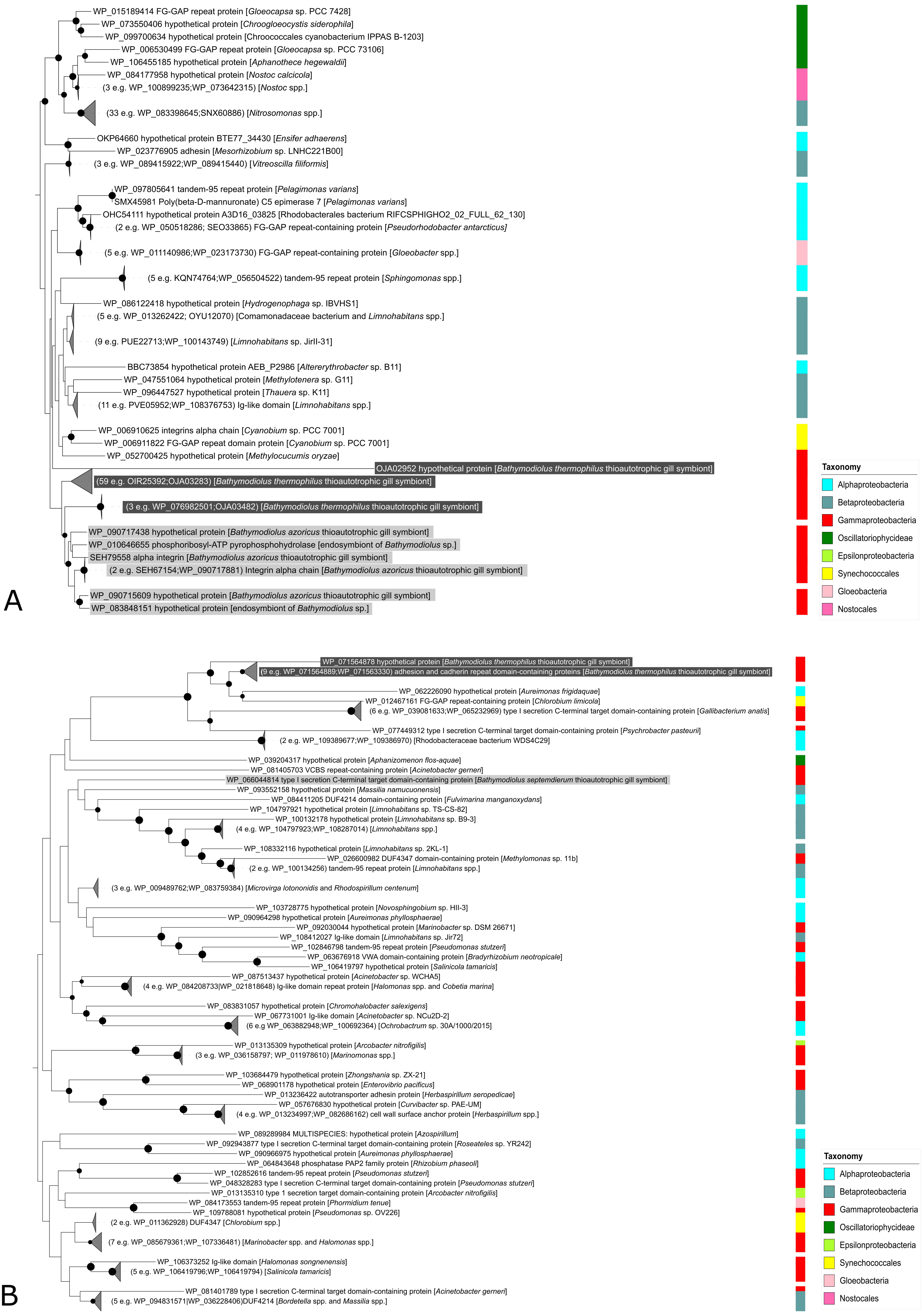

Supplementary Figure S1: Phylogeny of two *B. thermophilus* symbiont attachment-related proteins (ARPs), an integrin (**A**) and an adhesin (**B**). The trees were reconstructed using maximum likelihood analyses with 100 bootstrap replicates, resulting from an alignment of 1,140 amino acid positions (A) and 3,751 amino acid positions (B), respectively. Circles represent branches with a bootstrap value >70 and the size is proportional to the support. Polytomies were introduced where branch points had a bootstrap support <20. *B. thermophilus* symbiont proteins are highlighted in dark grey, proteins of other thioautotrophic *Bathymodiolus* symbionts are highlighted in light grey. Note that branches for highly similar sequences of the same organism and function were collapsed into groups (wedges). In these cases, the total number of sequences in the respective group is given in parentheses with accession numbers for two representatives. For example, the *B. thermophilus* symbiont integrin OIR25392 (A) belongs to a group of 59 nearly identical *B. thermophilus* symbiont proteins, all of which are more similar to each other than to proteins of related bacteria.
