# Supplementary material for: Comparative proteomics of related symbiotic mussel species reveals high variability of host–symbiont interactions
Source: ISME J. 2019 Nov 4;14(2):649–56. doi: 10.1038/s41396-019-0517-6 (PMC6976577; doi:10.1038/s41396-019-0517-6)
Supplement: Supplementary file 3 — Supplementary Figure S2 [file 41396_2019_517_MOESM3_ESM.pdf]

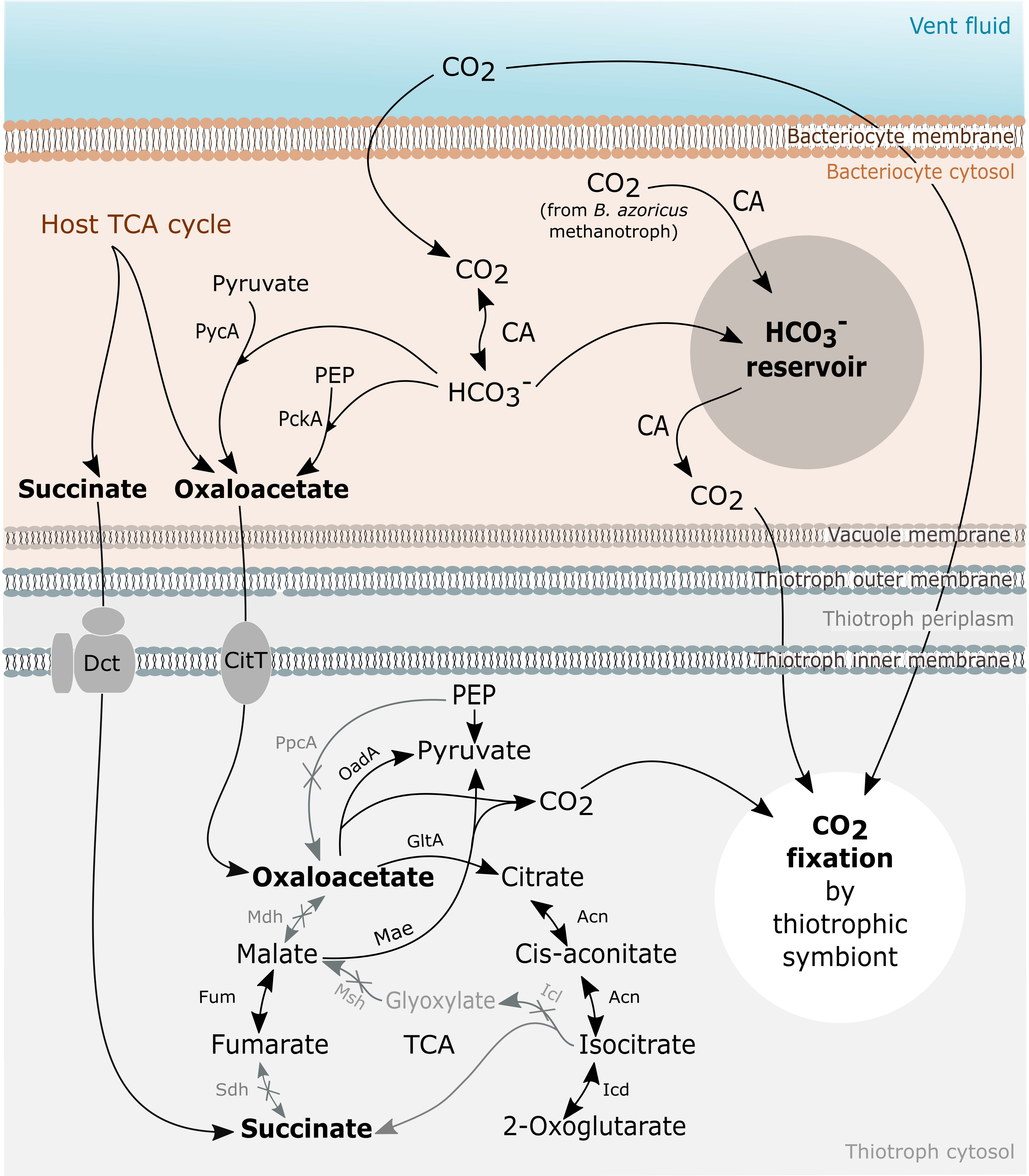

Supplementary Figure S2: Proposed model of CO_2 concentration in *Bathymodiolus*. The host enzyme carbonic anhydrase (CA) supposedly "traps" diffusible CO_2 by reversible conversion into non-diffusible HCO_3^- and thus creates a pool of immobilized CO_2 (HCO_3^- reservoir) in the gill tissue. In *B. azoricus*, this carbon pool could also be fed from CO_2 produced by the methanotrophic symbiont. Concentrated CO_2/HCO_3^- could then fuel both, carbon fixation by the thiotrophic symbiont, and oxaloacetate production by the host. The host produces oxaloactetate and succinate in the direct vicinity of the symbionts using the enzymes PycA (pyruvate carboxyltransferase) and PckA (phosphoenolpyruvate carboxykinase), which were particularly abundant in symbiont-cointaining samples. The host supposedly provides these intermediates to the thiotrophic symbionts. Many TCA cycle enzyme-encoding genes are missing in all four thiotrophic *Bathymodiolus* symbiont genomes analyzed in this study (grey crossed out arrows), suggesting that the symbionts cannot produce oxaloactetate and succinate autonomously. CitT: citrate transporter, Dct: tripartite ATP-independent periplasmic transporter, Mdh: malate dehydrogenase, Mae: malic enzyme, OadA: oxaloacetate decarboxylase, Icl: isocitrate lyase, Msh: malate synthase, PEP: phosphoenolpyruvate, Sdh: succinate dehydrogenase, PpcA: phosphoenolpyruvate carboxylase, Fum: fumarase, GltA: citrate synthase, Acn: aconitase, Icd: isocitrate dehydrogenase.
