# Supplementary material for: Comparative proteomics of related symbiotic mussel species reveals high variability of host–symbiont interactions
Source: ISME J. 2019 Nov 4;14(2):649–56. doi: 10.1038/s41396-019-0517-6 (PMC6976577; doi:10.1038/s41396-019-0517-6)
Supplement: Supplementary file 4 — Supplementary Figure S3 [file 41396_2019_517_MOESM4_ESM.pdf]

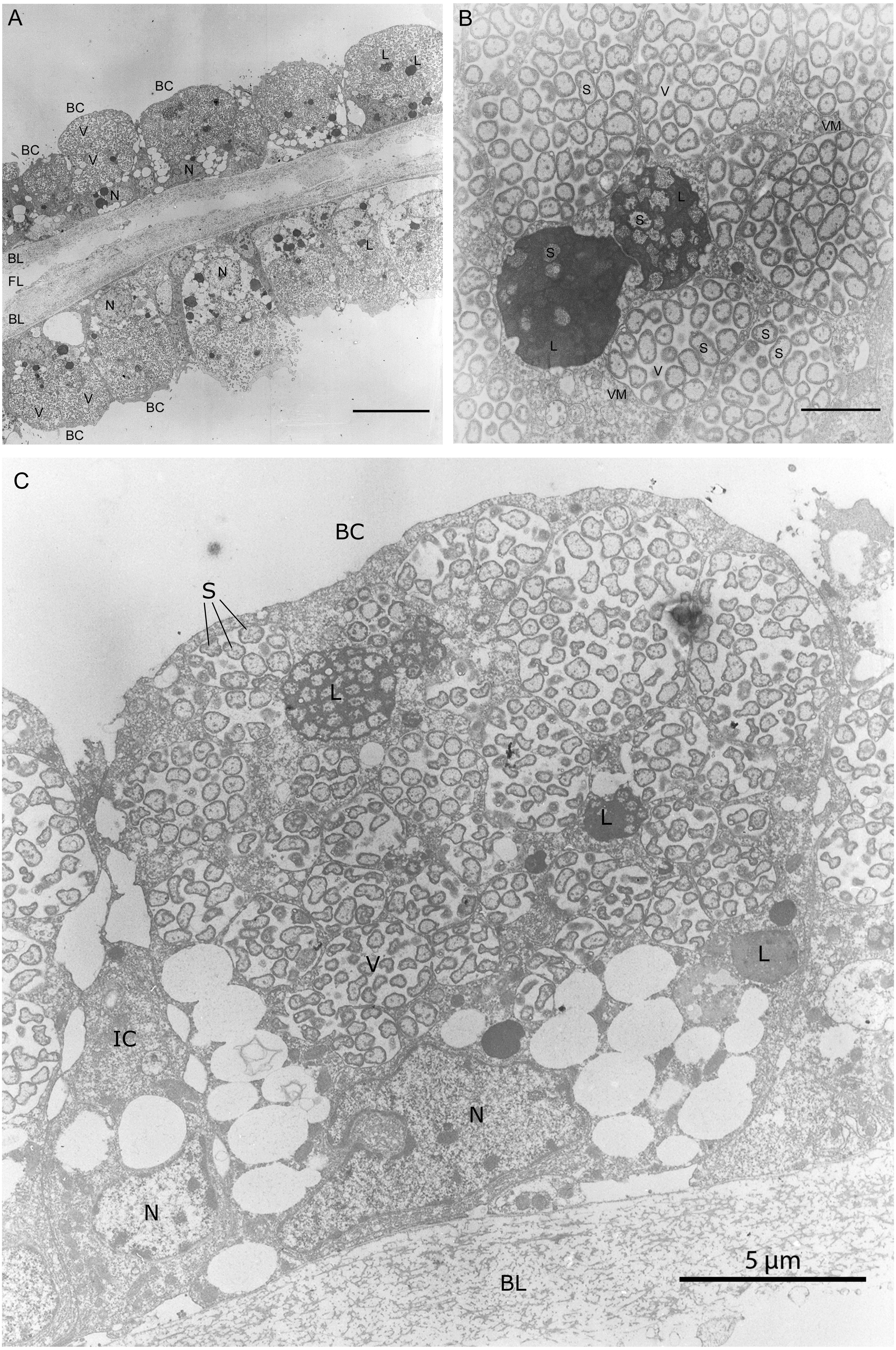

Supplementary Figure S3: Thiotrophic symbionts in *B. thermophilus* gill tissue sections. **A**: The transmission electron micrograph shows a single gill filament with central filament lumen (FL) enclosed by basal laminae (BL) and a unicellular layer of symbiont-containing bacteriocytes (BC). The symbionts are contained in host cell vacuoles (V) concentrated at the apical pole of the bacteriocytes (Fiala-Médioni et al., 1986). N: nucleus, L: lysosome. Scale bar: 15 µm. **B**: Lysosomal digestion of symbionts by the host. More than 20 symbiont cells (S) are enclosed in one vacuole, surrounded by a vacuole membrane (VM). Lysosomes (L) are visible among the symbiont-containing vacuoles. Some lysosomes contain symbiont cells, indicating that the host digests its symbionts. Scale bar = 2 µm. **C**: Close up of a single bacteriocyte located on a gill filament (detail of A). A thin symbiont-free intermediary cell (IC) is visble next to the voluminous bacteriocyte.
