# Supplementary material for: Comparative proteomics of related symbiotic mussel species reveals high variability of host–symbiont interactions
Source: ISME J. 2019 Nov 4;14(2):649–56. doi: 10.1038/s41396-019-0517-6 (PMC6976577; doi:10.1038/s41396-019-0517-6)
Supplement: Supplementary file 5 — Supplementary Figure S4 [file 41396_2019_517_MOESM5_ESM.pdf]

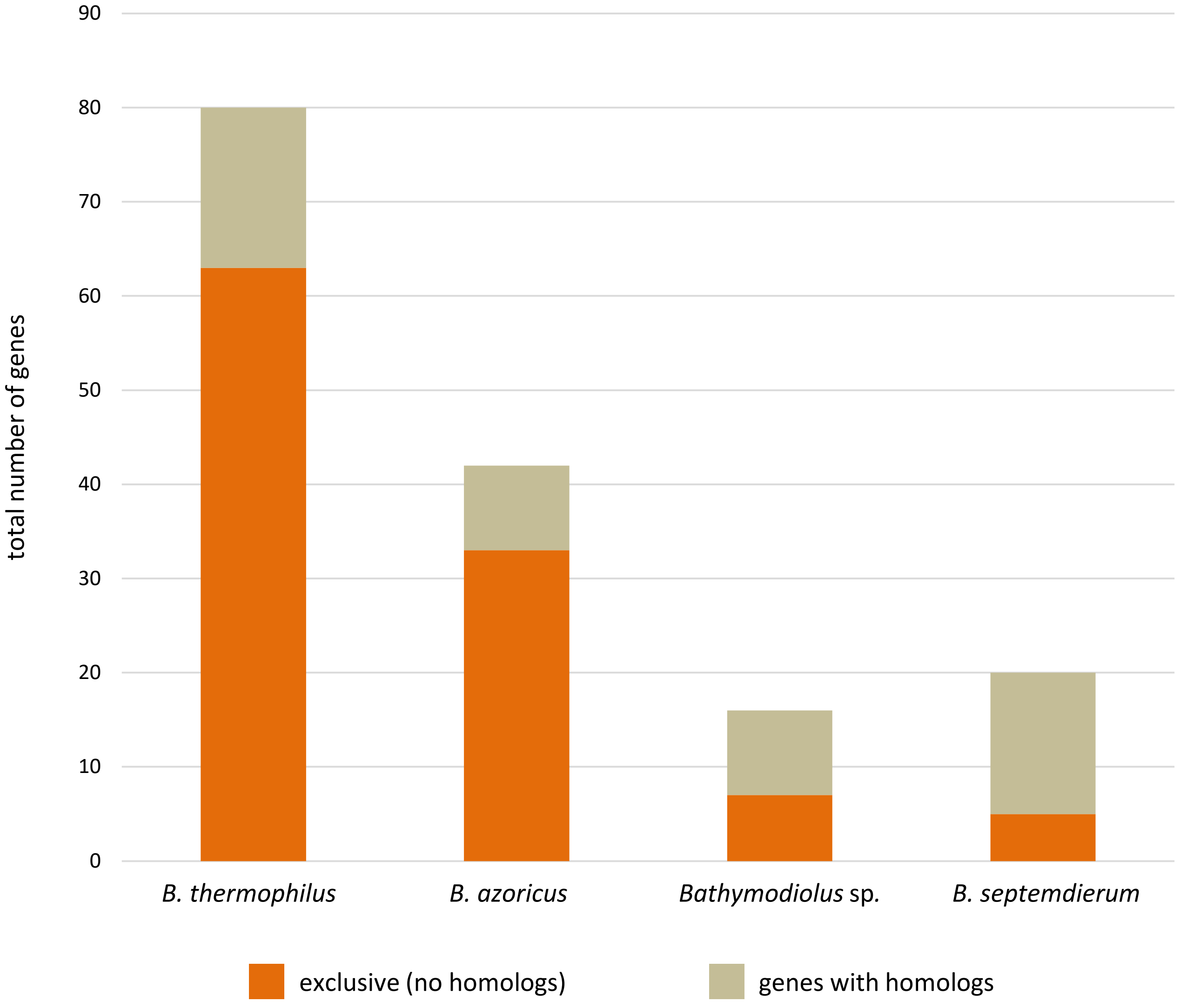

Supplementary Figure S4: Phage defense-related genes in thiotrophic *Bathymodiolus* symbionts. Total numbers of CRISPR-Cas and restriction-modification-related genes in the genomes of symbionts from four hosts (X-axis) were compared. Grey: genes that have homologs in one or several of the other genomes in comparison. Orange: genes that are exclusive to this genome (no homologs in the other related symbiont genomes). See Supplementary Table S7e for all gene functions.
