# Supplementary material for: Comparative proteomics of related symbiotic mussel species reveals high variability of host–symbiont interactions
Source: ISME J. 2019 Nov 4;14(2):649–56. doi: 10.1038/s41396-019-0517-6 (PMC6976577; doi:10.1038/s41396-019-0517-6)
Supplement: Supplementary file 6 — Supplementary Figure S5 [file 41396_2019_517_MOESM6_ESM.pdf]

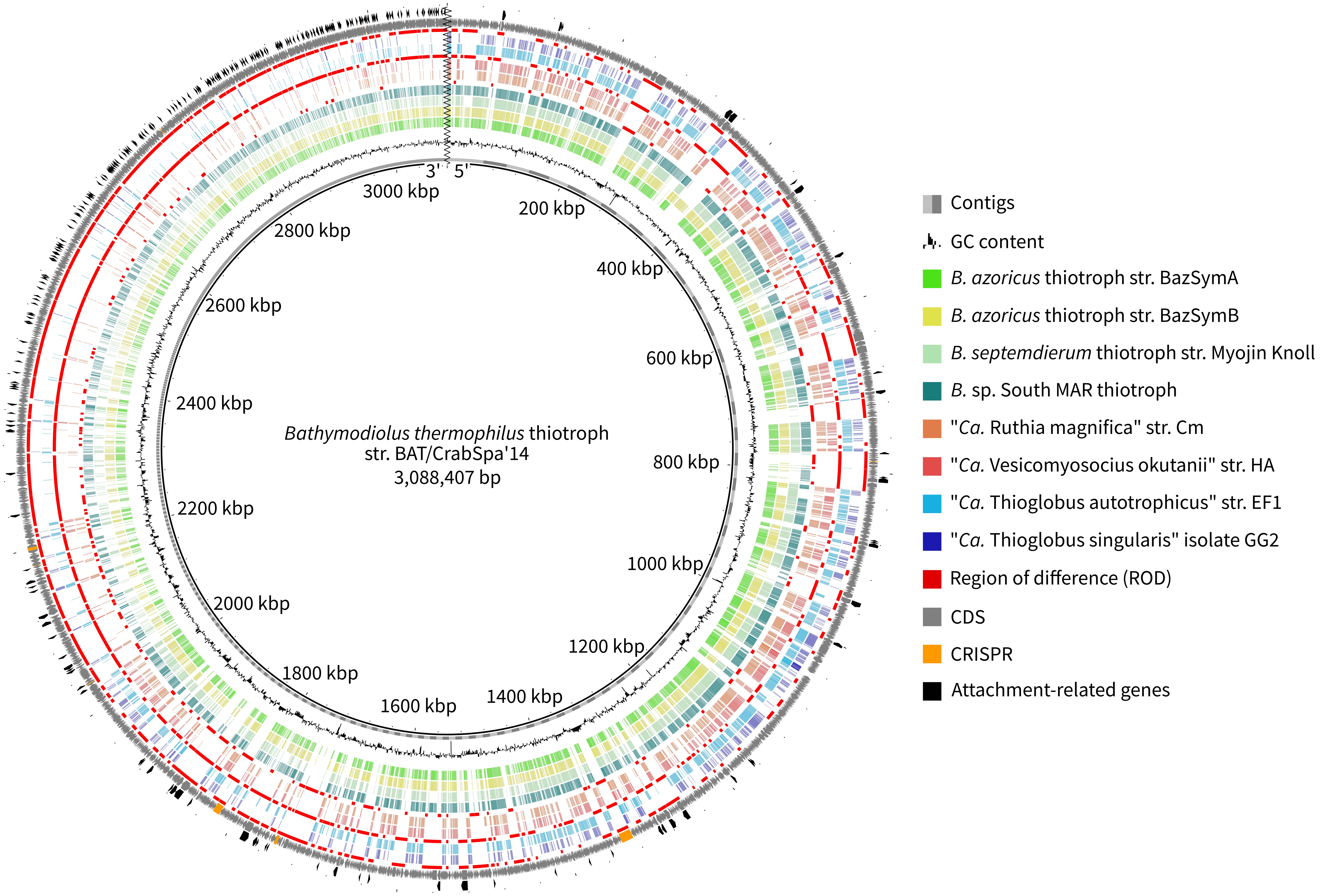

Supplementary Figure S5: Genome comparison of the *B. thermophilus* symbiont and related thiotrophic bacteria. The draft genome of the *B. thermohilus* thiotroph str. BAT/CrabSpa'14 (center) was sorted by contig size and compared by BLASTN analysis („-task blastn -evalue 2e-10 -dust no", see Supplementary Methods for details). The inner circles show the contig boundaries (alternating silver/gray) and GC content (black), respectively, in the *B. thermophilus* symbiont's genome. Colored concentric circles indicate identical genome regions in other thiotrophic *Bathymodiolus* symbionts (i.e., thiotrophic symbionts of *B. azoricus* (strains BazSymA/B), *B. septemdierum* (str. Myojin Knoll) and *Bathymodiolus* sp. South MAR), in giant clam symbionts („*Ca*. R. magnifica" str. Cm and „*Ca*. V. okutanii" str. HA), and in free-living relatives („*Ca*. T. autotrophicus" str. EF1 and „*Ca*. T. singularis" isolate GG2). Bright red arcs encircling each of these three groups of genomes represent the group's "regions of difference" (RODs) in relation to the *B.thermophilus* symbiont genome as a reference. Arrows in the outer circles show the *B. thermophilus* symbiont's coding sequences (CDS, grey), CRISPR regions (orange) and genes encoding attachment-related proteins (ARPs, black). Note that most of the *B. thermophilus* symbiont's ARP genes (black arrows in outermost circle) are located in RODs, i.e., these genes are not encoded in the respective other genomes in comparison.
