# Supplementary material for: Comparative proteomics of related symbiotic mussel species reveals high variability of host–symbiont interactions
Source: ISME J. 2019 Nov 4;14(2):649–56. doi: 10.1038/s41396-019-0517-6 (PMC6976577; doi:10.1038/s41396-019-0517-6)
Supplement: Supplementary file 7 — Supplementary Table S1 [file 41396_2019_517_MOESM7_ESM.pdf]

Supplementary Table S1a: Overview of all samples analyzed in this study. Samples were measured in LTQ-Orbitrap Classic or LTQ-Orbitrap Velos mass spectrometers. Numbers of biological replicates are indicated for all sample types. *two biological replicates were pooled into one sample for MS analysis; sol: soluble protein fraction; mem: enriched membrane-associate protein fraction.

		B. thermophilus		*B. azoricus*	
sample type		LTQ-Orbitrap Velos	LTQ-Orbitrap Classic	LTQ-Orbitrap Velos	LTQ-Orbitrap Classic
enriched symbionts (gradient pellet)	sol	3	-	2	3
	mem	-	-	-	2*
gill tissue	sol	3	-	2	-
	mem	-	3	-	2*
enriched host fraction (supernatant)	sol	-	-	2	-
foot tissue	sol	3	-	2	-

Supplementary Table S1b: Genomes of *Bathymodiolus* symbionts and related thiotrophic bacteria used for comparative genomics in our study. NCBI GenBank or RefSeq accession number and completeness of the genome are indicated. *IMG Taxon ID.

	Site of collection	Genome assembly / GenBank accession	Complete-ness	Reference
Thiotrophic *Bathymodiolus* symbionts				
B. thermophilus thiotroph (BATSym)	Tica, EPR (9°50.39′N, 104°17.49′W)	GCF_001875585.1 / MIQH00000000.1	96%	Ponnudurai *et al* ., 2017
B. azoricus thiotroph (BAZSymA)	Menez Gwen, MAR (37°45′35′′N, 31°38′15.6′′W)	GCF_001298715.1 / CDSC00000000.2	97%	Sayavedra *et al* ., 2015
B. azoricus thiotroph (BAZSymB)	Menez Gwen, MAR (37°45′35′′N, 31°38′15.6′′W)	GCA_001298695.2 / CVUD00000000.2	90%	Sayavedra *et al* ., 2015
Bathymodiolus sp. thiotroph (BspSym)	Lilliput, SouthMAR (09°32′50.9′′S, 13°12′33.3′′W)	CAEB00000000.1/ 2518645510*	95%	Petersen *et al* ., 2011
B. septemdierum thiotroph (BSEPE)	Myojin Knoll, Japan (32°1041′N, 139°2193′E)	GCF_001547755.1 / AP013042	closed	Ikuta *et al* ., 2015
Thiotrophic clam symbionts				
"*Ca.* Ruthia magnifica"	EPR (9°N)	GCF_000015105.1 / CP000488	closed	Roeselers *et al* ., 2010
"*Ca.* Vesicomyosocius okutanii"	Sagami Bay (35°06′60.00′′N, 139°22′59.99′′E)	GCF_000010405.1 / AP009247	closed	Kuwakara *et al* ., 2007
Free-living thiotrophic relatives				
"*Ca.* Thioglobus autotrophicus" (str. EF1)	isolate from oxygen mimum zone (lab cultivated strain)	GCA_001293165.1 / CP010552	closed	Shah and Morris, 2015
"*Ca.* Thioglobus singularis" (strain PS1)	isolate from oxygen mimum zone (lab cultivated strain)	GCF_001281385.1 / CP008725	closed	Marshall and Morris, 2015

Supplementary Table S1c: Components of the protein database used in this study to identify *B. thermophilus* and *B. azoricus* host and symbiont proteins. Amino acid sequences from available *Bathymodiolus* thiotrophic and methanotrophic symbiont genomes were compiled together with the *B. azoricus* EST database to create a single comprehensive database (see Experimental Procedures). *These EST bins were obtained by clustering those sequences that belong to the thiotrophic and methanotrophic symbionts from the *B. azoricus* EST library based on sequence homology. **http://transcriptomics.biocant.pt/deepSeaVent/

Target organism	Sequence database (reference)	Database source (project ID)
Thiotrophic symbionts of *B. thermophilus* and *B. azoricus*	*B. azoricus* thiotroph sequences from *B. azoricus* EST library* (Bettencourt *et al* ., 2010)	DeepSeaVent database**
	B. azoricus thiotroph assemblies BazSymA and BazSymB (Sayavedra *et al,.* 2015)	NCBI (PRJEB8263 and PRJEB8264)
	Bathymodiolus sp. thiotroph assembly BspSym (Sayavedra *et al* ., 2015)	NCBI (PRJNA65421)
	B. thermophlus thiotroph assembly BATSym (Ponnudurai *et al* ., 2017)	NCBI (PRJNA339702)
Methanotrophic symbiont of *B. azoricus*	*B. azoricus* methanotroph sequences from *B. azoricus* EST library* (Bettencourt *et al* ., 2010)	DeepSeaVent database**
	B. azoricus methanotroph assembly BazMethA (Ponnudurai *et al.,* 2016)	NCBI (PRJEB13769)
	Bathymodiolus sp. methanotroph assembly BspMeth (Ponnudurai et al., 2016)	NCBI (PRJEB13047)
B. thermophilus and *B. azoricus* hosts	*B. azoricus* host sequences from *B. azoricus* EST library* (Bettencourt *et al* ., 2010)	DeepSeaVent database**

Supplementary Table S1d: Number of protein identifications obtained in different sample types of *B. thermophilus* and *B. azoricus* . Besides whole gill and foot tissue, enriched symbiont fractions (Sym) and host fractions (Host) were analyzed (see Experimental Procedures for details). In addition to the soluble protein fractions (all sample types), membrane protein fractions (Mem) were analyzed for gill samples and enriched symbiont samples. MS/MS measurements were performed using LTQ-Orbitrap Velos and LTQ-Orbitrap Classic mass spectrometers. For an overview of all measurements and replicate numbers see Supplementary Table S1a.

		LTQ-Orbitrap Velos				LTQ-Orbitrap Classic			
		Sym	Host	Foot	Gill	Sym	Gill Mem	Sym Mem	*All samples*
B. thermophilus									
Thiotrophic symbiont	identified proteins total	1,353		11	879		756		*1,523*
	% of all identified proteins in sample	58.9%		1.0%	44.8%		42.5%		*43.8%*
Host	identified proteins total	945		1,080	1,084		1,022		*1,951*
	% of all identified proteins in sample	41.1%		99.0%	55.2%		57.5%		*56.2%*
	Grand total:	*2,298*		*1,091*	*1,963*		*1,778*		*3,474*
B. azoricus									
Thiotrophic symbiont	identified proteins total	808	279	76	469	783	440	390	*1,154*
	% of all identified proteins in sample	33.0%	15.3%	4.9%	20.9%	34.0%	26.1%	31.7%	*25.2%*
Methanotrophic symbiont	identified proteins total	329	157	20	233	233	127	112	*441*
	% of all identified proteins in sample	13.5%	8.6%	1.3%	10.4%	10.1%	7.5%	9.1%	*9.6%*
Host	identified proteins total	1,308	1,383	1,444	1,538	1,285	1,119	728	*2,977*
	% of all identified proteins in sample	53.5%	76.0%	93.8%	68.7%	55.8%	66.4%	59.2%	*65.1%*
	Grand total:	*2,445*	*1,819*	*1,540*	*2,240*	*2,301*	*1,686*	*1,230*	*4,572*
